# Identifying Sequence Effects on Chain Dimensions of Disordered Proteins by Integrating Experiments and Simulations

**DOI:** 10.1021/jacsau.4c00673

**Published:** 2024-11-14

**Authors:** Andrea Holla, Erik W. Martin, Thomas Dannenhoffer-Lafage, Kiersten M. Ruff, Sebastian L. B. König, Mark F. Nüesch, Aritra Chowdhury, John M. Louis, Andrea Soranno, Daniel Nettels, Rohit V. Pappu, Robert B. Best, Tanja Mittag, Benjamin Schuler

**Affiliations:** † Department of Biochemistry, University of Zurich, Winterthurerstrasse 190, 8057 Zurich, Switzerland; ‡ Department of Structural Biology, St. Jude Children’s Research Hospital, 262 Danny Thomas Place, Memphis, Tennessee 38105, United States; § Laboratory of Chemical Physics, National Institute of Diabetes and Digestive and Kidney Diseases, National Institutes of Health, Bethesda, Maryland 20892-0520, United States; ∥ Department of Biomedical Engineering and Center for Biomolecular Condensates, Washington University in St. Louis, St. Louis, Missouri 63130, United States; ⊥ Department of Biochemistry and Molecular Biophysics, Center for Biomolecular Condensates, Washington University in St. Louis, St. Louis, Missouri 63130, United States; # Department of Physics, University of Zurich, Winterthurerstrasse 190, 8057 Zurich, Switzerland

**Keywords:** intrinsically disordered proteins, single-molecule spectroscopy, Förster resonance energy transfer (FRET), atomistic simulations, coarse-grained simulations, chain dimensions, local expansion and compaction

## Abstract

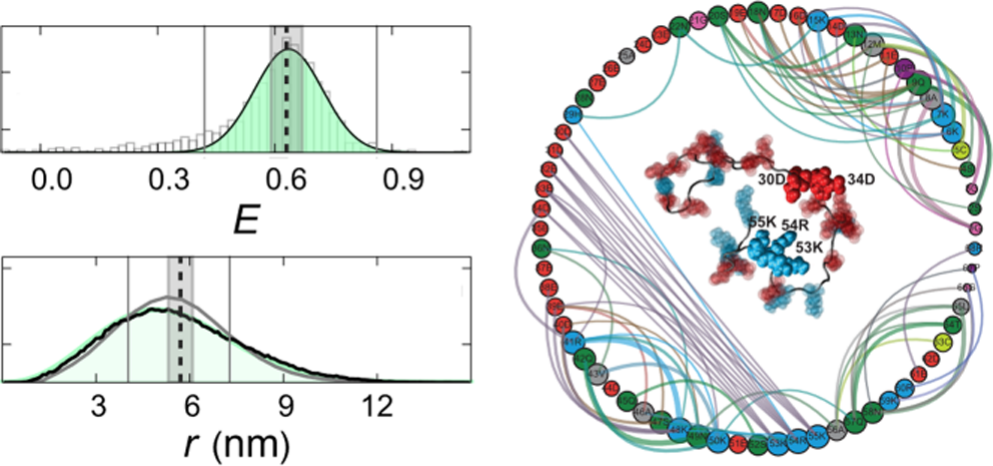

It has become increasingly evident that the conformational distributions of intrinsically disordered proteins or regions are strongly dependent on their amino acid compositions and sequence. To facilitate a systematic investigation of these sequence-ensemble relationships, we selected a set of 16 naturally occurring intrinsically disordered regions of identical length but with large differences in amino acid composition, hydrophobicity, and charge patterning. We probed their conformational ensembles with single-molecule Förster resonance energy transfer (FRET), complemented by circular dichroism (CD) and nuclear magnetic resonance (NMR) spectroscopy as well as small-angle X-ray scattering (SAXS). The set of disordered proteins shows a strong dependence of the chain dimensions on sequence composition, with chain volumes differing by up to a factor of 6. The residue-specific intrachain interaction networks that underlie these pronounced differences were identified using atomistic simulations combined with ensemble reweighting, revealing the important role of charged, aromatic, and polar residues. To advance a transferable description of disordered protein regions, we further employed the experimental data to parametrize a coarse-grained model for disordered proteins that includes an explicit representation of the FRET fluorophores and successfully describes experiments with different dye pairs. Our findings demonstrate the value of integrating experiments and simulations for advancing our quantitative understanding of the sequence features that determine the conformational ensembles of intrinsically disordered proteins.

## Introduction

Large parts of the proteomes of higher eukaryotes consist of intrinsically disordered proteins (IDPs), which do not adopt a well-defined three-dimensional structure under physiological conditions.^1^ For instance, ∼58% of human proteins contain both folded domains and intrinsically disordered regions (IDRs).^2^ IDRs occur in a variety of structural contexts, from tails and linkers between folded domains to fully disordered proteins, and they are particularly prevalent in regulation, such as in transcription and signaling,^3^ as well as in cellular organization via phase separation.^4^ Despite the lack of a well-defined tertiary structure, however, the conformational properties of IDPs are far from uniform: They range from compact states that can be rich in secondary structure to less compact ensembles all the way to highly expanded chains with no detectable secondary structure.^5−16^

For classifying and quantifying this continuous spectrum of disorder, concepts from polymer physics can be useful.^3,14,17,18^ For instance, based on the combination of net charge per residue and fraction of charged residues, IDPs can be grouped into strong and weak polyelectrolytes and polyampholytes,^19^ and classified by their chain dimensions in terms of ensemble-averaged quantities, such as their hydrodynamic radius, radius of gyration, or end-to-end distance.^6,12,18,20,21^ A helpful quantity for characterizing the dimensions of unfolded and disordered proteins independent of chain length is the scaling exponent, ν,^9,22^ which relates the chain dimensions, *R*, to the number of residues or chain segments, *N*, as *R* ∝ *N*^ν^. For infinitely long homopolymers, ν can take values of 1/3 for compact globules (and globular folded proteins), 1/2 for Flory random coils, and ∼0.588 for excluded volume chains.^22^ However, intermediate and larger values are commonly observed in simulations and experiments.^9,16,23−25^ Examples are highly charged sequences with pronounced electrostatic repulsion,^9,14,26^ which can approach ν ≈ 1 for rod-like conformations.^27^ Other reasons for deviations from canonical scaling are finite-size effects^28,29^ and heterogeneous patterns of intrachain interactions owing to the heteropolymeric nature of IDPs,^12,30^ especially the contributions of high fractions of charged residues^7−9^ and charge patterning.^10^

Considerable effort has been made to relate the dimensions of IDPs to their sequence properties and enable a predictive understanding of how intrachain interactions determine the sizes and shapes of the IDPs. Emerging consensus suggests that sequences rich in hydrophobic residues and certain polar tracts tend to favor compaction, whereas sequences rich in charged residues and proline tend to be more expanded.^3,14−16,18,31−35^ Polyelectrolytes dominated by a single type of charge are most expanded,^7,8,31^ whereas the attraction of opposite charges in polyampholytes can lead to compaction or long-range structural preferences depending on the patterning of oppositely charged residues.^7,10,11,36^ The use of coarse-grained models parametrized based on experimental results^15,37−40^ has enabled steps toward the analysis of conformational distributions across entire proteomes.^16,25^ However, the systematic quantitative assessment of sequence contributions and the parametrization of IDP models is still complicated by the heterogeneity of molecular systems that have been studied experimentally, which usually vary both in length and sequence composition, and are investigated under disparate solution conditions. To furnish a data set that avoids such limitations, we thus selected naturally occurring IDRs of identical lengths but with very different sequence properties and probed their intrachain distances by single-molecule Förster resonance energy transfer (FRET). In selected cases, we used complementary experimental methods, especially small-angle X-ray scattering (SAXS) for quantifying chain dimensions and NMR spectroscopy for identifying residue-specific intrachain interactions. We analyzed the results using atomistic simulations based on the ABSINTH model^41^ to identify main determinants of chain dimensions and used the data to optimize a coarse-grained IDP model with an explicit representation of the fluorophores.

## Results

**Figure 1:**
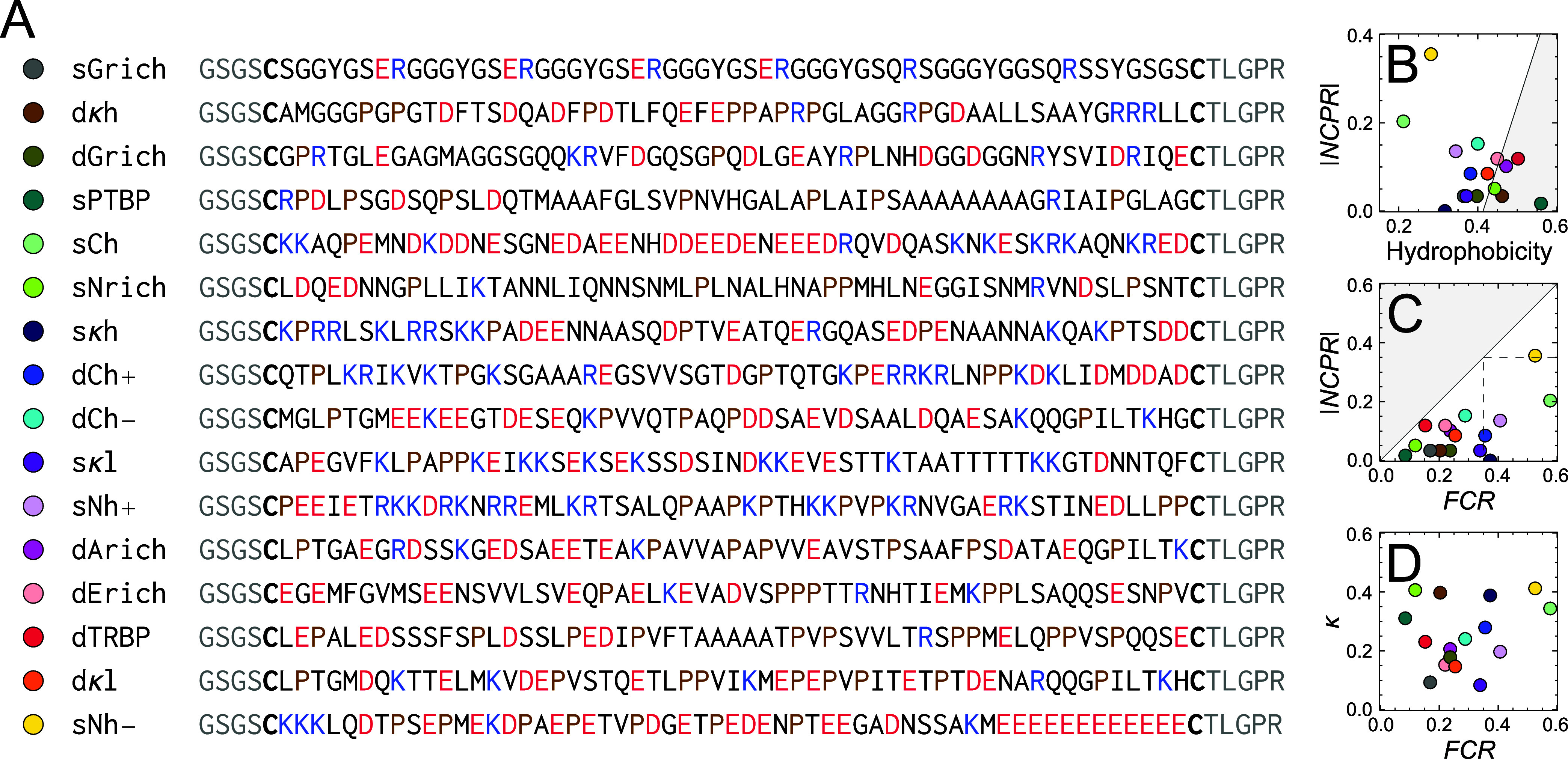
Sequences and sequence properties of the selected IDRs. (A) Selected sequences of IDRs, with acidic amino acids shown in red, basic residues in blue, Pro residues in brown, and Cys residues used for labeling in bold. The residues terminal of Cys (gray) are a result of the cloning/expression strategy used. Those residues and Cys were not included for calculating sequence parameters. (B–D) Average sequence properties cover a wide range. Hydrophobicity, net charge per residue (*NCPR*), and fraction of charged residues (*FCR*) are normalized by the total number of residues in each sequence. (B) The line indicates the separation of IDRs and folded proteins (gray region) suggested by Uversky et al.^47^ (C) Most selected IDRs fall in the region of weak polyampholytes and polyelectrolytes with *FCR* ≤ 0.35 and |*NCPR*| ≤ 0.35. IDRs with *FCR* > 0.35 and |*NCPR*| ≤ 0.35 are considered strong polyampholytes, and those with *FCR* > 0.35 and |*NCPR*| > 0.35 strong polyelectrolytes^19^ (dashed lines). The gray region cannot be populated. (D) The charge pattering metric κ describes the distribution of charged amino acids along the chain.^10^

We selected 16 IDRs, each comprising 57 residues, from the linker regions connecting folded domains in RNA-binding proteins (Figure 1A, Figure S1). The large number of known RNA-binding proteins^42^ allows for a wide variety of available sequences with very different physicochemical properties, while at the same time ensuring that the selected sequences are biologically relevant in terms of their amino acid composition. The sequence conservation of IDRs in RNA-binding proteins^43^ attests to their functional importance beyond tethering of the folded domains. Their functions are possibly related to posttranslational modifications, interactions with the folded domains or RNA, or the optimal spacing of domains resulting from the sequence-encoded chain dimensions of the IDRs.^44^ By scoring the corresponding linker sequences available in UniProtKB^45^ for average hydrophobicity, net charge, fraction of charged residues, charge patterning, and amino acid composition (Figure S1), we identified examples that maximize the diversity of these properties (Figure 1B–D), from low to high hydrophobicity; from low to high net charge; from polyelectrolytes to polyampholytes; from low to high charge segregation; and including examples enriched in individual amino acids, such as Gly, Glu, Ala, and Asn (see the Materials and Methods section for details). Only sequences with a disorder score in Metapredict^46^ above 0.5 along the entire sequence were selected (Figure S2). Far-UV circular dichroism spectra of the recombinantly produced IDRs confirm the absence of pronounced secondary structure (Figure S3). The remaining differences between the spectra are suggestive of sequence-specific contributions to the conformational ensembles, but they are difficult to analyze quantitatively. Taken together, we have thus identified a set of naturally occurring IDRs of identical length that cover a broad spectrum of the key parameters commonly used to assess the properties of disordered proteins.

### Chain Dimensions Vary Widely among Sequences

**Figure 2:**
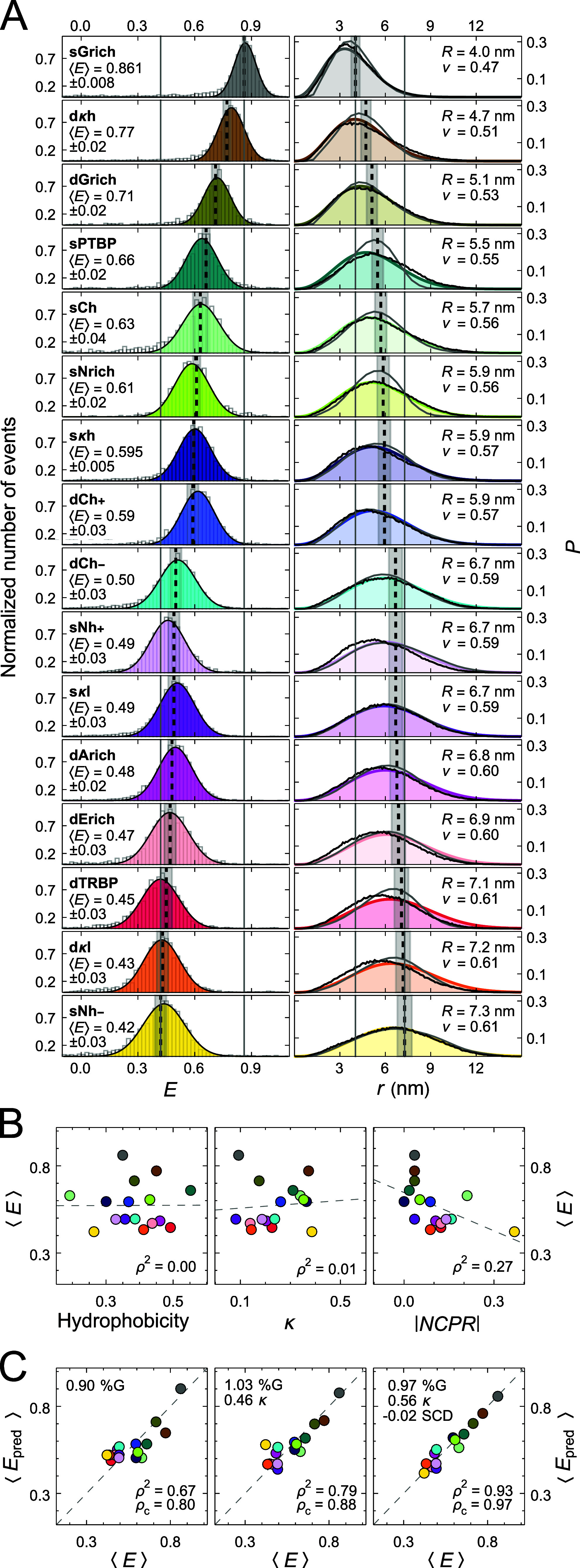
Chain dimensions from single-molecule FRET and correlations with sequence parameters. (A, left) Transfer efficiency histograms of Cy3B/CF660R-labeled IDRs (gray) fit with Gaussian peak functions (color). The average of the mean transfer efficiency, ⟨*E*⟩, from at least three experiments is indicated by a black dashed line, with the standard deviation shown as a gray band. Vertical gray lines indicate the lowest and highest average ⟨*E*⟩ observed across the series. (A, right) Distance distributions based on the SAW-ν model^26^ (colored line), reweighted ensembles from ABSINTH^41^ simulations (gray line), and from an optimized coarse-grained model (black line). Vertical dashed lines indicate the average root-mean-squared distance (*R*) from SAW-ν, with a gray error band based on a systematic uncertainty of ±7% in the Förster radius.^39,53^ Values for the average *R* and average ν are shown in the top right corners. Vertical gray lines indicate the largest and smallest values of *R* across the series. (B) Correlations between different sequence parameters and average transfer efficiency. The dashed line and the coefficient of determination, ρ^2^, were obtained from linear regression. (C) Multiple linear regression was used to identify combinations of sequence parameters that maximize the correlation with transfer efficiency. The regression coefficients for each of the sequence parameters used as regressors (%*G*, κ, and *SCD*) are indicated. The dashed line is the identity line, and ρ_c_ is the concordance correlation coefficient. Color code for the sequences is given in Figure 1.

The selected IDR sequences, bracketed with Cys residues for fluorophore labeling via maleimide chemistry, were expressed recombinantly, purified, and labeled with donor and acceptor dyes for single-molecule FRET. By working at picomolar protein concentrations in single-molecule measurements, we could avoid aggregation and phase separation, even for sequences with low solubility that are exceedingly difficult to investigate with ensemble experiments at high concentrations. Moreover, by resolving conformational subpopulations in single-molecule experiments, species such as small aggregates (which may go unnoticed in ensemble measurements) can be detected^48^ and prevented by optimized sample handling or separated out in the analysis (Figure S4). We performed multiparameter confocal single-molecule measurements using pulsed interleaved excitation^49^ with all 16 labeled IDRs and identified a single transfer efficiency peak under our experimental conditions for each sequence. The results reveal a remarkably broad range of intramolecular transfer efficiencies from ∼0.4 to ∼0.9 for the different IDRs despite their identical chain lengths (Figure 2A), reflecting the pronounced dependence of the chain dimensions on amino acid sequence.

We had previously observed that the charge of the fluorophores needs to be accounted for to quantitatively explain the dimensions of IDPs with simple polymer models.^7,9^ We thus used two different FRET pairs with different net charges to assess such effects. One widely used pair comprises the dyes Alexa 488 and Alexa 594 (Förster radius *R*_0_ = 5.4 nm), each of which carries a net charge of −2; the other pair comprises the dyes Cy3B and CF660R^50^ (*R*_0_ = 6.0 nm), which carry a net charge of 0 and −1, respectively (Figure S5). We find that protein sequences rich in basic residues yield lower average intramolecular distances when labeled with the more negatively charged Alexa pair, whereas other sequences yield very similar results for both dye pairs (Figure S6A). Similarly, SAXS measurements of the Alexa 488-labeled IDR dCh– showed the increase in *R*_g_ expected from the addition of the fluorophore compared to unlabeled dCh–, but for sNh+ the increase was much smaller (Figure S6B). NMR spectroscopy confirmed the presence of more attractive interactions between positively charged residues and the Alexa fluorophores than with Cy3B and CF660R (Figure S6C,D). The large range of transfer efficiencies and chain dimensions we observe is robust with respect to the dye pair used, but to minimize the influence of the dyes on the FRET-based assessment of chain dimensions, we focus on the results obtained with Cy3B/CF660R.

Using the SAW-ν model, a semiempirical approximation with an adjustable length scaling exponent^26^ to infer intramolecular distance distributions for the different sequences, we obtained root-mean-squared end-to-end distances, *R*, between 4.0 and 7.3 nm (Figure 2A), corresponding to almost a factor of 2 between the most compact (sGrich) and the most expanded IDR (sNh-), and a factor of ∼6 in chain volume. The inferred average scaling exponents,^26^ ν, are between 0.47 to 0.61, corresponding to the range from effective theta conditions to excluded volume chains.^18,22^ Although a detailed interpretation of these scaling exponents is complicated by finite-size effects^29^ and the contributions from heterogeneous interaction patterns within heteropolymers,^12,30^ they imply that these IDRs are rather open chains and more expanded than a compact globule. However, it is worth noting that the two Gly-rich sequences sGrich and dGrich are among the most compact of the set, suggesting an important role for Gly in chain compaction.

It may not be surprising that highly charged sequences rank among the most expanded chains,^7,8^ and correspondingly, the average net charge per residue (*NCPR*) shows a correlation with the observed transfer efficiency (Figure 2B). Average Kyte–Doolittle hydrophobicity^51^ shows remarkably little correlation with transfer efficiency, which is likely to be connected to the requirement of alternative hydrophobicity scales to describe protein phase separation.^38,40^ Similarly, the transfer efficiency shows little correlation with the charge patterning parameter κ, which only applies to sequences with high fractions of charged residues^52^ (Figure 2B). However, sequence composition clearly influences the chain dimensions; examples of individual residues whose content in the sequences correlates with transfer efficiency with a coefficient of determination of ρ^2^ ≥ 0.36 are Gly (favoring compaction), Arg (favoring compaction), Val (favoring compaction), Thr (favoring expansion), and Pro (favoring expansion) (Figure S6).

In view of these correlations, we thus asked how well the transfer efficiencies correlate with combined compositional biases. For instance, multiple linear regression combining the fraction of Gly and κ as regressors yields ρ^2^ = 0.79, i.e., 79% of the variance in the observed transfer efficiencies can be accounted for with this combination alone. Combining the fraction of Gly with κ and sequence charge decoration,^11^*SCD*, yields an even higher ρ^2^ value of 0.93 (Figure 2C). However, based on a leave-one-out analysis to identify the dominant contributions (Figure S7), we find that individual members of the data set have a large effect on the result; for instance, including sGrich greatly improves the ρ^2^ for linear regressions that account for the fractions of Gly or Tyr. These correlation analyses clearly show an important effect of sequence composition on chain dimensions and can thus provide interesting clues about which residues or sequence characteristics are relevant. However, the set of IDRs investigated here cannot provide sufficiently broad sampling of sequence space to uniquely define chain dimensions based on regression analysis alone. We thus turned to simulations for a more detailed analysis.

### Atomic-Level Characterization of Conformational Ensembles from Simulations

**Figure 3:**
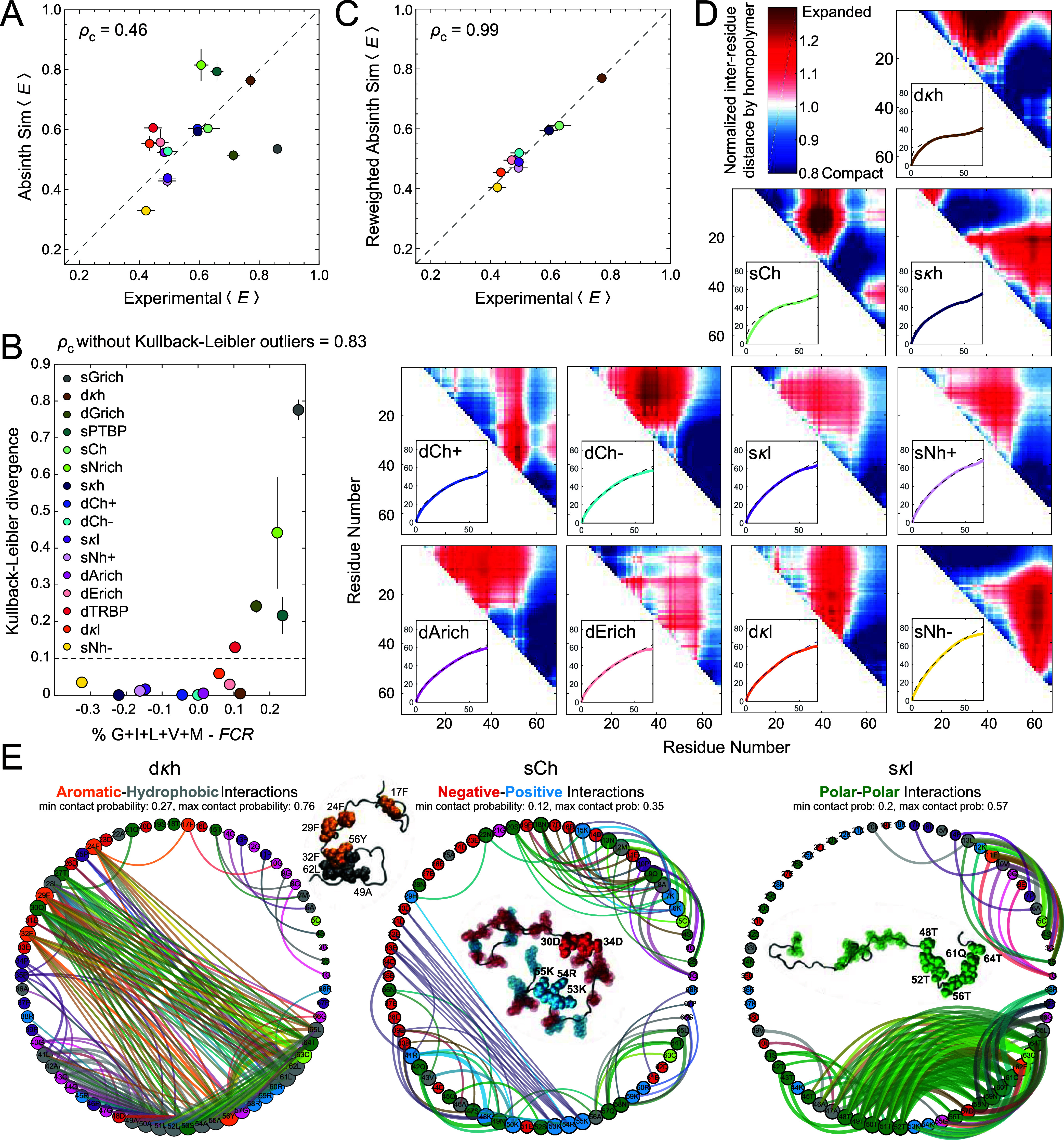
ABSINTH simulations provide atomic-level characterizations of conformational ensembles. (A) Correlation between the mean transfer efficiencies, ⟨*E*⟩, from experiment and unweighted ABSINTH simulations (ρ_c_: concordance correlation coefficient). (B) Kullback–Leibler divergence quantifying the deviations between the unweighted (prior) and reweighted (posterior) ensembles obtained with ABSINTH, plotted as a function of the fraction of Gly and hydrophobic residues minus the fraction of charged residues (*FCR*) of the sequences. Minimal deviations have a KL divergence of ≤0.1. Based on this cutoff, five of the 16 sequences, characterized by high Gly or high aliphatic content and lower charge content, require substantial reweighting (sPTBP, dTRBP, sGrich, sNrich, and dGrich). (C) Correlation between ⟨*E*⟩ from experiment and reweighted ABSINTH simulations for sequences with KL divergence <0.1. (D) Average inter-residue distances from reweighted ABSINTH simulations (minimum spacing of 10 amino acids), relative to the value from the best fit of a homopolymer model (see color scale) determined by *R*_*i*,*j*_ = ⟨*r*_*i*,*j*_^2^⟩^1/2^ = *A*_0_|*i* – *j*|^ν^, where *r*_*i*,*j*_ is the spatial distance between residues *i* and *j*, *A*_0_ is an adjustable prefactor that reports on the chain persistence length, |*i* – *j*| is the linear sequence separation between residues *i* and *j*, and ν is the scaling exponent. Regions of local expansion relative to the equivalent homopolymer are shown in red, and areas of local compaction are in blue. The insets show ensemble-averaged inter-residue distances, ⟨*R*_*i*,*j*_⟩ = ⟨⟨*r*_*i*,*j*_^2^⟩^1/2^⟩ (in Å), versus |*i* – *j*| (colored line). The best homopolymer fit is shown as a dashed gray line. Here, the double average implies averaging over all pairs of residues *i* and *j* that are |*i* – *j*| apart in the sequence (the outer average), and the spatial separations, *R*, between specific pairs of residues across all conformations in the ensemble (the inner average). Only IDRs with a KL divergence ≤0.1 are shown. (E) Contact networks illustrate different interaction preferences for different IDRs. Residues are shown as nodes, with the circle size related to the mean contact probability between that residue and all other residues more than two residues away in sequence. Edges are drawn between two residues if their contact probability is at least 35% of the maximum contact probability observed for that IDR. Specifically, edges are shown for mean contact probabilities between 0.27 and 0.76, 0.12 and 0.35, and 0.2 and 0.57 for dκh, sCh, and sκl, respectively. The width of an edge is 10 times the mean contact probability. Here, a contact distance of 10 Å is used, such that charge interactions can be observed. Gly is shown in pink, Ser, Thr, Asn, and Gln in green, Arg, Lys, and His in blue, Asp and Glu in red, Phe, Trp, and Tyr in orange, Met, Val, Ile, Leu, and Ala in gray, Pro in purple, and Cys in lime green. Edge colors are mixtures of interacting residue colors. Representative snapshots are visualized using VMD^62^ and chosen by finding the frame that has the highest weight with a radius of gyration (*R*_g_) within 0.5 Å of the average *R*_g_ for the IDR. Contact networks for the remaining proteins are shown in Figure S10.

Key discoveries regarding sequence-ensemble relationships of IDPs have been made using atomistic simulations based on the ABSINTH implicit solvation model and force field paradigm.^41^ In the ABSINTH model, polypeptides and solution ions are represented in atomistic detail, and the aqueous solvent is modeled implicitly. Measured free energies of solvation serve as a benchmark against which solvation inhomogeneities are calibrated. These inhomogeneities are gleaned by using solvent-accessible volumes, and the changes to solvation are balanced by changes to the effective charge, which is an efficient way of capturing dielectric inhomogeneities. To compare end-to-end distances from ABSINTH simulations to those inferred from FRET measurements, an atomistic representation of the fluorophores was included based on a rotamer library that takes into account dye configurations that are sterically allowed (see the Materials and Methodssection). To compare FRET efficiencies from simulations and measurements (Figure 3A), we computed the concordance correlation coefficient^54^ (ρ_c_), which combines correlation (precision) and deviation from perfect concordance (accuracy), yielding ρ_c_ = 0.46 for the ABSINTH ensembles.

Overall, ABSINTH captures certain overall trends from the FRET measurements, but there are clear deviations from the experimental results. To better understand where ABSINTH fails and succeeds with the current data set, the conformational ensembles were reweighted, as described previously^12^ (Figure 3C). We then compared the unweighted (prior) ensembles to the reweighted (posterior) ensembles. This analysis allowed us to assess whether there were specific sequence features that mandate substantial reweighting of the ABSINTH-derived ensembles when comparing the computed and measured FRET efficiencies. The results of this analysis are presented in terms of the Kullback–Leibler (KL) divergence between the unweighted and reweighted ensembles (Figure 3B), which is below 0.06 for 11 of the 16 sequences and greater than 0.1 for five of the sequences. The largest divergences result for the two Gly-rich sequences and the N-rich sequence. The two sequences with large fractions of aliphatic residues also show KL divergences above 0.1. Omitting the sequences with KL divergences above 0.1 from the analysis yields ρ_c_ = 0.83 for the unweighted and ρ_c_ = 0.99 for the reweighted ensembles (Figure 3C).

The most compact IDR observed experimentally, sGrich, contains several stretches rich in Gly. Water is a poor solvent for polyglycine,^20,55^ polyglutamine,^6^ and other types of polar tracts.^56^ The preference of Gly-^57^ and Gln-rich sequences^58^ for collapsed conformations and their low solubility has been predicted and computed using all-atom simulations^59^ with different types of force fields,^60^ and observed experimentally.^20^ The challenges arise in ABSINTH for Gly- and Asn-rich sequences because of the delicate interplay between favorable hydration of the polar backbone and side chains and the favorable intrachain interactions between polar groups, even though ABSINTH does not have a challenge with Gln-rich or Gln- and Asn-rich sequences.^61^

Before analyzing the reweighted ensembles in detail, we computed three different parameters that quantify the ensemble-averaged global sizes and shapes for each of the IDRs using the ABSINTH-derived prior and posterior ensembles (Figure S8). First, we quantified the correspondence for the global radius of gyration (*R*_g_). Overall, the deviations are minimal, with the two largest outliers being the Gly-rich sequences (Figure S8). Next, we computed how the overall shape changes upon reweighting by computing the ensemble-averaged asphericity (Figure S8). Here, we observed a few more deviations compared to *R*_g_, but the general consistency in compaction and expansion suggests that the sequence controls the local interactions and deviations from a homopolymer. Finally, we compared the root-mean-squared end-to-end distances, *R*, for unweighted versus reweighted ensembles. We find that the sequences with high KL divergence fall outside the 95% confidence interval. Across the three parameters, the deviation between unweighted and reweighted ensembles is the largest when we compare *R*.

The preceding analysis suggests that robust inferences regarding conformational ensembles can be drawn from either the reweighted or unweighted ABSINTH simulations for 11 out of the 16 sequences, which we analyze in more detail (Figure 3D). We quantified the scaling of average inter-residue distances (⟨*R*_*i*,*j*_⟩ = ⟨⟨*r*_*i*,*j*_^2^⟩^1/2^⟩) between residues *i* and *j* with an alternative approach to describing sequence separation (|*i* – *j*|) (insets, Figure 3D). To determine how well the conformational ensembles can be described by homopolymer models, the standard polymer relationship ⟨*R*_*i*,*j*_⟩ = *A*_0_|*i* – *j*|^ν^ was fit to extract *A*_0_ and ν, the apparent scaling exponent (insets in Figure 3D, dashed gray line). Although the value of ν is not meaningful for internal scaling profiles that show plateauing behavior, this type of comparison can still be helpful to determine whether there are nonuniform interactions along a chain. Therefore, we examined the distance between residues normalized by the best-fit homopolymer models (Figure 3D). All IDRs show deviations from the uniform length scaling expected for homopolymers, as shown by regions along the sequence of compaction (blue) or expansion (red) compared to the IDR-specific homopolymer model. Additionally, the degree of compaction or expansion relative to an equivalent homopolymer reference can vary along the sequence (Figure 3D, Figure S9), highlighting the heterogeneity of interactions within each sequence. Overall, these results suggest that even though global features, such as end-to-end distance, can be correlated with sequence composition, additional analyses from atomistic simulations can provide detailed insights into the heteropolymeric properties of the IDRs.

### Optimizing a Coarse-Grained IDP Model with Explicit Fluorophores

An alternative approach to describing sequence-ensemble relationships is to use the experimental data to variationally optimize the parameters of the simulation model itself.^15,39^ Coarse-grained implicit solvent models, which have only a limited set of adjustable parameters, are naturally suited to this approach.^15,16,37,39,40^ In this case, the amino acids are represented by beads with the appropriate volumes and charges, whose interactions are accounted for via a screened Coulomb potential and a residue-specific short-range potential that represents interactions such as hydrophobicity or hydrogen bonding in addition to the excluded volume. Force field parameters for such models have previously been optimized based on statistical potentials and/or by comparison with experimental data.^37,38,40^ However, in general, the experimental data employed have been collected under different solution conditions (e.g., different temperatures, pHs, salt concentrations), complicating their coherent use for model refinement. Data sets comprising a large sequence diversity of IDRs measured under identical solution conditions are therefore expected to be useful for benchmarking and refining simulation models and parameters that are transferable to a wide range of IDPs.

We thus employed the experimental results from our 16 IDRs to identify the residue-specific short-range interaction parameters for a hydrophobicity scale (HPS) model^38,63^ that best describe the entire data set. To this end, we use the values of Tesei et al.^40^ as a starting point and employ the force balance approach,^38,64,65^ where the short-range interaction parameters λ are iterated to optimize agreement between the simulated and experimental transfer efficiencies. A particular advantage of this method is that the fluorophores and their interactions with the rest of the sequence can be included in the model explicitly and parametrized with the same strategy. This approach thus enables interactions of the fluorophores to be taken into account that go beyond the excluded volume effects most commonly considered in accessible volume^66,67^ and rotamer library^50,68−71^ schemes. We chose a dye representation that reflects the size, structure, and charge distribution of the different fluorophores and consists of charged, uncharged, and dye linker beads (Figure S5).

**Figure 4:**
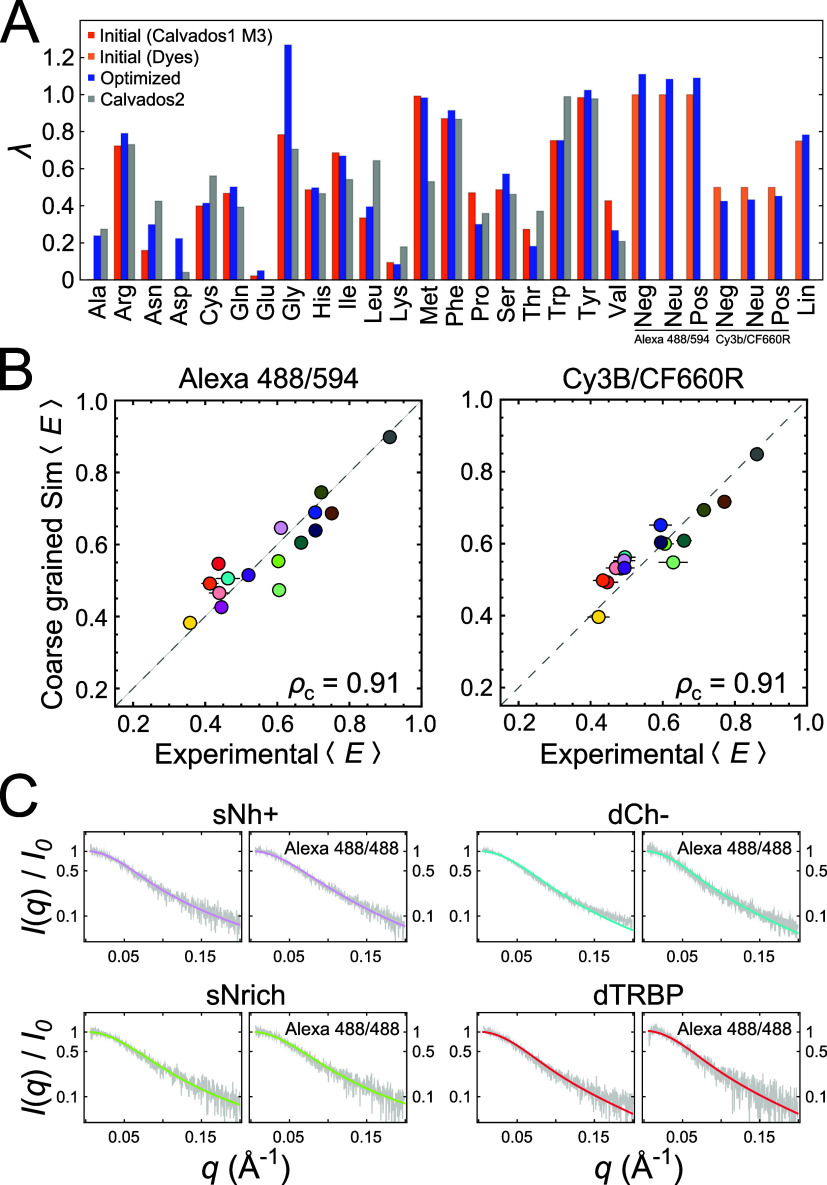
Coarse-grained simulations consistently describe amino acid and dye interactions. (A) Initial (orange for amino acids and light orange for dyes), optimized (blue), CALVADOS 2^72^ (gray) values of the short-range interaction parameter (λ) for each amino acid, dye (Alexa 488/594 and Cy3B/CF660R; Neg: negatively charged, Neu: neutral, Pos: positively charged), and dye linker (Lin) beads. Note that the initial values for Ala and Asp are too small to be visible on this scale (Table S3). (B) Correlation between the mean transfer efficiencies from experimental data and from resulting coarse-grained simulations for both dye pairs used. The dashed line is the identity line, and ρ_c_ is the concordance correlation coefficient. (C) SAXS curves of selected IDRs (gray lines) compared to results based on the simulations (colored lines). For each IDR, results for unlabeled protein are shown in the left plot, and results for protein labeled with Alexa 488 at both Cys residues are shown in the right plot. The simulation value at a *q* of 0 was used for normalization of both experiments and simulations.

With the optimized parameters for the HPS model (Figure 4A), we obtained correlations with ρ_c_ = 0.91 between experiment and simulation for the IDRs labeled with either dye pair (Figure 4B), compared with ρ_c_ = 0.70 and 0.64 before optimization. The short-range interaction parameters for the amino acids after optimization are reasonably close to the starting values, which were obtained previously based on experimental data and hydrophobicity scales of amino acids;^40^ indeed, the new parameters yield similar results when benchmarked against the original CALVADOS training set^40^ (Figure S11). The increased value for Gly reflects the pronounced compaction of Gly-rich sequences we observe experimentally, and the increased values for Arg and Tyr are in line with previous results suggesting an important role of these residues for chain compaction and phase separation.^16,40,73−76^ We note, however, that while the CALVADOS parameters slightly underestimate compaction for Gly-rich sequences in the present data set, our HPS parameters slightly overestimate compaction for the longer Gly-rich sequences in the CALVADOS training set, a conflict that likely points to limitations of the form of the HPS model itself. Importantly, by essentially treating the fluorophores as part of the sequence, both the results for the Alexa dye pair and Cy3B/CF660R can be described well. Although a detailed rationalization of the resulting short-range interaction parameters for the fluorophores based on their chemical structure is challenging at this level of coarse-graining, the larger values for the Alexa dyes are in accord with the stronger dye-peptide interactions observed in the SAXS and NMR data compared to Cy3B/CF660R (Figure S6).

We further tested the optimized HPS parameters by calculating scattering curves from coarse-grained simulations of several IDRs with and without fluorophores and comparing with SAXS measurements of labeled and unlabeled sNh+, dCh–, sNrich, and dTRBP, and we found reasonable agreement (Figure 4C), further validating the model. As additional tests on independent sequences, we used previously published SAXS data on a series of IDPs^12^ and a mutationally destabilized variant of the β-helix protein PNt labeled with zero, one, or two copies of Alexa 488;^77^ overall, the data are well described by our model. In the case of PNt, the results indicate a very moderate decrease in radius of gyration upon attaching one or two dyes (although limitations in SAXS data quality yield a large uncertainty for the double-labeled variant, Figure S12A). For the same PNt variant with Alexa 488 and 594 attached (at the same sites as for the PNt variant doubly labeled with Alexa 488^77^), the measured transfer efficiency (⟨*E*⟩ = 0.6; Figure S12B) is reasonably reproduced by the HPS model (⟨*E*⟩ = 0.52). Altogether, the optimized coarse-grained model thus provides a residue-specific way of quantifying the dimensions of disordered proteins. Moreover, the fluorophores can be incorporated and their interactions parametrized within the same framework, treating them essentially as an additional set of residues, so that the effect of the FRET dyes on chain dimensions can be predicted and distances and distance distributions between the dyes can be obtained directly from the simulations and compared to experiment.

Finally, we compared end-to-end distance distributions resulting from the three different methods employed: the analytical SAW-ν polymer model, the optimized coarse-grained HPS model, and the reweighted atomistic ABSINTH simulations (Figure 2A). The distributions are similar in all cases, indicating the consistency of the different approaches at the level of the overall chain dimensions. For a more detailed comparison, we calculated distance maps from the HPS simulations and the reweighted ABSINTH simulations (Figure S13). This analysis reveals three groups of sequences. For nine of the 16 sequences, we find strong positive correlations between the normalized distance maps for the reweighted ABSINTH and HPS models (Pearson correlation coefficient ρ > 0.6); for four of the 16 sequences, weak positive correlations (0.3 < ρ < 0.5); and for three of the sequences—sGrich, sPTBP, and sNrich—we observe anticorrelation (ρ < 0). These are also the IDRs for which extensive reweighting was required for the ABSINTH ensembles (Figure 3B), but for most of the IDRs, the intrachain interactions predicted by the models are similar. The discrepant cases highlight the challenges associated with the interplay between chain-solvent and intrachain interactions in arriving at a consistent description of ensembles for Gly- and Asn-rich sequences,^61^ and for sequences where secondary structural preferences in the ABSINTH model cause deviations, such as sPTBP. Indeed, the circular dichroism spectra of sPTBP and some other sequences (Figure S3) show hints of residual secondary structure.

## Discussion

We investigated a set of 16 IDRs selected from linker sequences of naturally occurring proteins with identical lengths but very different sequence compositions to probe the sequence dependence of the conformational ensembles of disordered proteins. Notably, since all sequences investigated here originate from the linker regions between RNA-binding domains, their chain dimensions may have been an evolutionary factor contributing to the average distance between the domains^44^ and their interaction with RNA. The experimental results, consisting of single-molecule FRET efficiencies measured with two different dye pairs and complemented with SAXS and NMR, serve as a benchmark and provide an opportunity for systematically refining simulation models and force field parameters. Here, we tested and compared three approaches at very different levels of coarse-graining for modeling the conformational ensembles of 16 IDRs.

Analytical homopolymer models can be useful for inferring overall distance distributions and effective length scaling exponents; although they cannot provide details on heterogeneities in local compaction or expansion along the sequence, they are a simple and useful way of interpreting experimental data in terms of distance distributions,^17,18^ but their predictive power is limited. Simple correlations between chain dimensions and sequence composition also offer useful indications of the effect of individual residues on chain compaction, but simulations provide much more detail regarding the heteropolymer properties of disordered proteins. Our application of two different simulation approaches demonstrates the complementarity of atomistic and coarse-grained models. All-atom implicit solvent simulations using ABSINTH^41,61,78^ in combination with ensemble reweighting^12^ enable detailed analyses of residue-specific intrachain interaction networks that affect chain compaction and the resulting deviations from simple homopolymer models (Figure 3). Coarse-grained models facilitate the optimization of force field parameters to arrive at a transferable model, illustrated here with the HPS model^38,40,63^ combined with the force balance approach^38,64,65^ (Figure 4). We further show how FRET dyes can be incorporated and parametrized explicitly in the HPS model to achieve agreement between results using different fluorophores (Figure 4A,B). Overall, our work thus illustrates the mutual benefit of experiment and simulations: experimental data enable the testing and refinement of simulation models, and simulations enable a detailed structural interpretation of the experimental results.

## Conclusions

Our results demonstrate that the dimensions of IDRs exhibit a pronounced dependence on amino acid sequence. This result is consistent with a broad range of previous observations,^7−10,13,14,16,18,19,24,31,34,79−81^ but a noteworthy feature of the current work is that we focused on sequences of identical length, thus ensuring that differences in sequence-ensemble relationships do not arise from additional structure or sequence context. The measurements were performed under identical solution conditions, such as buffer, salt concentration, and pH, which greatly simplifies their direct quantitative comparison. Furthermore, the sequences were selected from natural proteins to ensure the biological relevance of their sequence compositions and to represent a broad range of sequence characteristics, which allows many types of effects to be accounted for. The analysis of the results using polymer models and both all-atom and coarse-grained simulations consistently shows that the chain dimensions and conformational properties cover a very broad range. Particularly pronounced contributions to chain compaction come from the content in Gly and aromatic residues; contributions to chain expansion come from charge repulsion and Pro residues. However, we did not identify a simple single descriptor that captures global chain dimensions, but compaction can be driven by different types of interactions in different sequences. From our data, we have derived a coarse-grained model that can be used to predict how such interactions affect the dimensions of other disordered proteins.

## Materials and Methods

### Sequence Selection and Characterization

To identify disordered protein regions, UniProtKB was searched for proteins containing at least two double-stranded RNA-binding domains,^82^ and sequences of interdomain linkers were identified that were 50–200 amino acids in length. In order to increase the compositional diversity of the sequences, a second pool of sequences was generated from proteins containing at least two RNA recognition motifs (RRMs).^83^ All sequences were characterized in terms of the following sequence properties. Normalized hydrophobicity was calculated using the scale of Kyte and Doolittle, which assigns a relative hydrophobicity index, *H*_*i*_, between −4.5 and +4.5 to each amino acid^51^
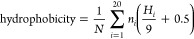
Here, *N* is the total number of amino acids in the sequence and *n*_*i*_ is the number of each of the 20 amino acid types within the polypeptide chain. The normalized hydrophobicity may adopt values between 0 and 1. The fraction of charged residues, *FCR*, and the net charge per residue, *NCPR*, were calculated according to Das and Pappu^10^ as
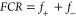

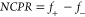


where *f*_+_ and *f*_–_ denote the fractions of positively and negatively charged residues, respectively. We calculate the charge patterning factor κ and sequence charge decoration^11^ (*SCD*) as described previously. A total of 16 linker sequences were selected to cover a large sequence parameter space. All sequences were shortened to 57 amino acids to rule out length-dependent effects in their comparison. In this process, care was taken to alter the average sequence properties as little as possible. Sequences containing Trp were excluded to minimize effects from dye quenching that can complicate quantitative analysis of FRET experiments.^84^ Two natural Cys residues were replaced by Ser in dErich, and sκl contains a spontaneous Ser to Ile exchange due to the instability of the gene in *Escherichia coli*. The naming of the IDRs was chosen to be suggestive of characteristic sequence properties (‘s’: derived from ssRNA binding proteins, ‘d’: derived from dsRNA binding proteins, ‘h’: high, ‘l’: low, ‘N’: net charge, ‘C’: charge, ‘+’: positively charged, ‘–’: negatively charged, “κ”: charge segregation, “Xrich”: enriched in amino acid X). All sequences are shown in Figure 1, and the UniProt codes of the source proteins and the sequence parameters are listed in Table S1.

Multiple linear regression was performed for all single, double, and triple combinations of the following 26 compositional features: Fraction A, D, E, G, K, L, N, P, Q, R, S, T, V, K+R, D+E, Polar, Aliphatic, Aromatic, and Chain Expanding, as well as *FCR*, |*NCPR*|, Hydrophobicity, Disorder Promoting, Isoelectric point, κ, and *SCD*. Compositional features were calculated using localCIDER.^85^ Fractions of C, F, H, I, M, W, and Y were not considered, as they each account for less than 2.5% of all residues in the linker IDR sequences. For Figure S7A, the ρ^2^ values are shown for all 26 single compositional features, all double combinations of compositional features with a ρ^2^ > 0.72, and all triple combinations of compositional features with a ρ^2^ > 0.855. The boxplots in Figure S7A show the distributions of ρ^2^ values for all 16 leave-one-out analyses.

### Protein Expression, Purification, and Fluorescence Labeling

Codon-optimized DNA sequences encoding the IDRs with two terminal Cys residues for site-specific dye labeling were purchased from GeneArt (Regensburg, Germany). Linker IDR sequences were cloned into a pET-20b(+) based plasmid (EMD Millipore), which contained an N-terminal His_6_-tag, as well as a C-terminal GB1 domain for improved expression fused to a His_6_-tag, both of which were separated from the IDR of interest via a thrombin cleavage site.^86^ For all constructs, thrombin cleavage resulted in a residual GSGSC overhang at the N-terminus and a CTLGPR overhang at the C-terminus of the protein.

The IDRs were expressed in *E. coli* Rosetta (DE3) cells (Merck Biosciences). Cultures were grown to an OD_600_ of 0.8 in LB medium containing carbenicillin, induced with 1 mM IPTG, and incubated at 20 °C overnight. For the preparation of isotope-labeled proteins, M9 minimal medium containing ^15^NH_4_Cl or ^15^NH_4_Cl and ^13^C_6_-glucose was used instead of LB medium. Cells were harvested, and pellets were resuspended in lysis buffer (100 mM NaH_2_PO_4_/Na_2_HPO_4_, 10 mM Tris-HCl, 6 M guanidinium chloride (GdmCl), 10 mM imidazole, 1 mM Tris(2-carboxyethyl)phosphine (TCEP), pH 8.0). Insoluble cell debris was removed by centrifugation. The soluble fraction was subjected to Nickel chelate affinity chromatography (Ni Sepharose excel, GE Healthcare Bio-Sciences). The lysis buffer was used for washing. For elution, the imidazole concentration was increased to 500 mM. Eluates were dialyzed against 50 mM Tris-HCl, 150 mM NaCl, and 10% (v/v) glycerol, pH 8.0, and the total protein concentration was quantified by measuring the absorbance at 280 nm (extinction coefficients: sGrich-GB1, 20,400 M^–1^ cm^–1^; dGrich-GB1, 12,950 M^–1^ cm^–1^; dκh-GB1, 11,460 M^–1^ cm^–1^; all others: 9970 M^–1^ cm^–1^). Subsequently, thrombin was added at 20 U/mg and the proteolytic digest was allowed to proceed for 1–3 h at room temperature. The reaction was quenched by adding 1 g/mL GdmCl. Protein solutions were then concentrated to a total volume of approximately 1 mL using Centriprep 3K centrifugal filter devices (EMD Millipore).

Protein samples were reduced by adding DTT at a final concentration of 10 mM and purified by reversed-phase high-performance liquid chromatography (RP-HPLC) on a C18 column (Reprosil Gold 200, Dr. Maisch GmbH) using 5% acetonitrile, 0.1% (v/v) trifluoroacetic acid (TFA) as buffer A and acetonitrile as buffer B. Eluates were lyophilized overnight and redissolved in 20 mM KH_2_PO_4_/K_2_HPO_4_, 6 M GdmCl, pH 7.3. Protein concentrations were quantified using a bicinchoninic acid (BCA) assay kit (Thermo Fisher Scientific Inc.) by measuring the absorbance at 562 nm. For fluorescence labeling, Alexa Fluor 488 C5 maleimide (Thermo Fisher Scientific Inc.) or maleimide-functionalized Cy3B (GE Healthcare AG) dissolved in anhydrous *N*,*N*-dimethylformamide (DMF) was added at a molar ratio of protein to dye of 1:0.7. The reaction was allowed to proceed at 4 °C overnight and quenched by the addition of DTT at a final concentration of 10 mM. Singly labeled protein was separated from unreacted and doubly labeled protein by RP-HPLC (see above), followed by lyophilization. Donor-labeled protein was redissolved in 20 mM KH_2_PO_4_/K_2_HPO_4_, 6 M GdmCl, pH 7.3. Alexa Fluor 594 C5 maleimide (Thermo Fisher Scientific, Inc.) or maleimide-functionalized CF660R (Biotium, Inc.) dissolved in anhydrous DMF was added in 3 times molar excess. The reaction was permitted to proceed as described above and quenched by adding DTT at a final concentration of 10 mM. Donor–acceptor labeled protein was purified by RP-HPLC (see above), lyophilized, and redissolved in 20 mM KH_2_PO_4_/K_2_HPO_4_, 6 M GdmCl, pH 7.3. Protein identity and site-specific labeling were confirmed by electrospray ionization mass spectrometry (ESI-MS). The reactivity of the two cysteine residues for the fluorophores is not identical, and some separation of labeling permutations was achieved during the purification of some IDRs, but in most cases, we used a mixture of permutants. In the case of sκh, where the dye permutants could be separated, the difference in their transfer efficiency was 0.02, indicating a minor effect on the results. Fluorescently labeled samples were stored at −80 °C until further use.

A synthetic codon-optimized N-terminal 334-amino acid segment of pertactin^77^ (PNt) with Cys residues in positions 29 and 117 was cloned into a pJ414 vector (ATUM) and transformed into *E. coli* BL21-DE3 cells (Agilent). Cells were grown at 37 °C in Luria–Bertani medium containing 100 μg mL^–1^ carbenicillin and induced for expression at an OD_600_ of 0.7 for 3 h. The cell pellet derived from 0.5 L of culture was suspended in 70 mL of buffer A [50 mM Tris-HCl, pH 8, 100 mM NaCl, 1 mM ethylenediaminetetraacetic acid (EDTA), 5 mM 2-mercaptoethanol, and 5 mM benzamidine], followed by the addition of lysozyme (100 μg mL^–1^) and sonicated at 4 °C. The insoluble recombinant protein was washed by resuspension in 70 mL of buffer A containing 1% Triton X-100 and subsequently in buffer A in the absence of Triton X-100. In all cases, the insoluble fraction was pelleted by centrifugation at 20,000*g* for 30 min at 4 °C. The final pellet was solubilized in 8 M urea, 50 mM Tris-HCl, pH 8.0, 5 mM EDTA, and 5 mM tris(2-carboxyethyl)phosphine (TCEP) and 1/4th of the protein (∼15 mg) was applied onto a Superdex-200 column (1.6 × 60 cm, Cytiva) equilibrated in 50 mM Tris-HCl, pH 8, 4 M GdmCl, 1 mM EDTA, and 1 mM TCEP at a flow rate of 1.4 mL min^–1^ at ambient temperature. Peak fractions with the highest purity were verified by mass spectrometry and used for the experiments. PNtCC was labeled with dyes as described for the other IDRs.

### Circular Dichroism Spectroscopy

Unlabeled constructs were dialyzed against 20 mM KH_2_PO_4_/K_2_HPO_4_, 1 mM DTT, pH 7.3, using Slide-A-Lyzer MINI Dialysis Devices, 3.5K MWCO (Thermo Fisher Scientific). Insoluble components were removed by centrifugation. Circular dichroism spectra from 190 to 250 nm were acquired on a spectropolarimeter (J-810, Jasco, or ChiraScan V100, Applied Photophysics) at 22 °C in quartz cells with a path length of 0.5 or 1 mm at concentrations of 0.1–0.5 mg/mL. Absorption data of those scans were used to determine the concentration of the peptides using their absorption at 214 nm.^87^

### Single-Molecule Spectroscopy

For single-molecule experiments, the donor–acceptor labeled IDRs were diluted to approximately 100 pM in 20 mM KH_2_PO_4_/K_2_HPO_4_, 125 mM KCl, pH 7.3 with 0.001% Tween 20, and 10 mM DTT for the Cy3B/CF660R-labeled or 147 mM 2-mercaptoethanol for the Alexa-labeled IDRs. The measurements were conducted at 22 °C using chambered cover slides (μ-Slide, ibidi). Different light sources were used for excitation, depending on the fluorophores used. For Alexa 488 excitation, an LDH-D-C-485 diode laser (PicoQuant GmbH) was employed. Alexa 594 and Cy3B excitation was achieved using a supercontinuum fiber laser (SC-450-4, Fianium Ltd.) filtered by a z582/15 or HC543.5/2 band-pass, respectively (Chroma Technology). CF660R was excited with an LDH-D-C-640 diode laser (PicoQuant GmbH). Lasers were operated at a pulse repetition rate of 20 MHz to achieve pulsed interleaved excitation of donor and acceptor.^88^ Fluorescence photons were collected with a UplanApo 60*x*/1.20W objective (Olympus) and passed through a suitable multiband mirror and a 100-μm confocal pinhole. Subsequently, photons were separated according to polarization by using a polarizing beam splitter and wavelength via suitable dichroic mirrors. Finally, photons were filtered by optical band-pass filters and detected by avalanche photodiodes. Photon arrival times were recorded with a HydraHarp 400 time-correlated single-photon counting system (PicoQuant) at a time resolution of 16 ps.

Photon bursts emitted by labeled IDRs diffusing through the confocal volume were identified as contiguous intervals of emission with interphoton times below 150 μs.^89^ FRET efficiency histograms shown in Figure 2A are based on a threshold of 50 photons per burst. Dual-channel-burst-search^90^ was applied to avoid artifacts from bleaching and blinking. Subsequently, bursts were corrected for differences in chromophore quantum yields, differences in detection efficiency of the detectors and spectral crosstalk obtained from measurements of free dye solutions, and direct acceptor excitation and background signal.^91^ The stoichiometry ratio^88,92^ of a photon burst was calculated according to
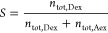
where *n*_tot,Dex_ and *n*_tot,Aex_ denote the corrected total number of photons emitted after donor or acceptor excitation, respectively. Bursts with 0.2 < *S* < 0.8 were used to calculate the transfer efficiency
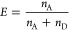
where *n*_D_ and *n*_A_ are the corrected donor and acceptor photon counts emitted upon donor excitation within a burst, respectively. Alternatively, the correction factors were inferred from the measurement of the IDRs with alternating excitation.^39,92^Figure 2 and Table S2 show the average of the mean transfer efficiencies of at least three independent measurements. The average standard deviations are ±0.014 or ±0.024 for the measurements using either the Alexa dye pair or Cy3B/CF660R, respectively, most of which were taken over the course of multiple years and on different instruments. Fluorescence polarization anisotropies were <0.1 for all samples and fluorophores, indicating that the orientational factor κ^2^ in Förster theory can be approximated by 2/3 due to rapid orientational averaging of donor and acceptor.^53^ Data analysis was performed using the Mathematica (Wolfram Research) package Fretica (https://github.com/SchulerLab).

### NMR Spectroscopy

All data were acquired on Bruker Avance 600 MHz spectrometers equipped with TCI triple-resonance cryogenic probes and pulsed-field gradient units. All spectra were referenced directly by using DSS for the ^1^H dimension; ^13^C and ^15^N frequencies were referenced indirectly. Samples were dissolved in a buffer identical to those used for smFRET measurements (20 mM KH_2_PO_4_/K_2_HPO_4_, 0.125 M KCl, 10 mM DTT, pH 7.3). For backbone assignment, the ^15^N, ^13^C isotopically labeled peptides were prepared to an approximate concentration of 0.5 mM. Standard 3D assignment experiments based on sensitivity-enhanced ^1^H, ^15^N HSQC (8 scans, 1024 × 256 complex data points) were collected. These included an HNCACB and CBCA(CO)NH (8 scans, 1024 (^1^H) × 32 (^15^N) × 128 (^13^C) complex data points, with 11, 24, and 70 ppm as ^1^H, ^15^N and ^13^C sweep width, respectively), an HN(CA)CO (8 scans, 1024 (^1^H) × 32 (^15^N) × 75 (^13^C) complex data points, with 11, 24, and 18 ppm as ^1^H, ^15^N, and ^13^C sweep widths), an HNCO (16 scans, 1024 (^1^H) × 32 (^15^N) × 75 (^13^C) complex data points, with 11, 32, and 22 ppm as ^1^H, ^15^N, and ^13^C sweep widths), and a HNCA (16 scans, 1024 (^1^H) × 32 (^15^N) × 95 (^13^C) complex data points, with 16, 25, and 30 ppm as ^1^H, ^15^N, and ^13^C sweep widths). Additionally, HN(CA)NNH (16 scans, 1024 (^1^H) × 32 (^15^N F1) × 60 (^15^N F2) complex data points, with 11, 24, and 24 ppm as ^1^H, ^15^N F1, and ^15^N F2 sweep widths) spectra provided connectivity between *i* and *i* ± 1 amide nitrogen nuclei. Data were processed using BRUKER Topspin version 3.4, NMRPipe^93^ (v.7.9) and analyzed using NMRfam SPARKY.^94^ HSQC spectra were acquired for the unlabeled and Cy3B-, CF660R-, Alexa 488-, and Alexa 594- labeled sNh+ (16 scans, 1024 × 256 complex data points) at an approximate concentration of 0.1 mM. Assignments were transferred to labeled sNh+ based on the assignments of the unlabeled protein. Broadening of the HSQC resonances was quantified using the ratio of the peak height of labeled protein to that of unlabeled. Chemical shift perturbation values were calculated as
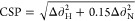


### Small-Angle X-ray Scattering

SAXS experiments were performed on the BioCAT (18-ID-D) beamline at the Advanced Photon Source at Argonne National Laboratory.^95^ Linker IDR samples were measured by coupling size-exclusion chromatography to a coflow X-ray sample chamber.^96^ In short, a 5/150 Superdex 75 increase column (Cytiva) was equilibrated in a buffer containing 20 mM KH_2_PO_4_/K_2_HPO_4_, 0.125 M KCl, pH 7.3, and 10 mM DTT. Elution of protein from the column was monitored by UV absorbance at 220 nm and integrated X-ray scattering intensity. Data reduction was performed at the beamline using the BioXTAS RAW software package.^97^ Subsequent analysis and averaging of SEC-SAXS data was performed using custom Matlab routines^98^ (Mathworks).

### Atomistic Simulations and Reweighting

Atomistic simulations of each of the IDRs were performed utilizing a homegrown adaptation of version 3 of the CAMPARI Monte Carlo simulation package (http://campari.sourceforge.net) and ABSINTH implicit solvation model and force field paradigm.^41,78^ For each sequence, five independent simulations were performed. The simulations use spherical droplets with radii of 150 Å. Simulations utilize a modified abs3.2_opls.prm parameter with explicit representations of ions,^99^ and the radii of sodium ions were set to 1.81 Å to avoid broken ergodicity due to ion chelation effects, especially around acidic groups. Neutralizing and excess Na^+^ and Cl^–^ ions were modeled explicitly, with an excess NaCl concentration of 20 mM. Simulations were performed at 340 K with 6.15 × 10^7^ steps, of which the first 1 × 10^7^ steps were taken as equilibration. The move set included translational, side chain rotation, concerted rotation, pivot, and proline puckering moves.^78^

For each replica, 1030 frames were saved and subjected to the addition of dyes using our in-house program COCOFRET. Briefly, for each frame 50 trials were attempted to attach Alexa 488 on the first Cys and attempts were discarded if the dye leads to steric classes with the IDR. Additionally, 50 separate trials were attempted to attach Alexa 594 to the second Cys and attempts were discarded if steric classes with the IDR exist. Attachment of dyes was performed by randomly selecting a rotamer from the HandyFRET rotamer library (http://karri.anu.edu.au/handy/rl.html) and making sure the γ-sulfur angles and bond lengths were ideal. Clashes were defined as any atoms within 5 Å of each other. Then, if at least 20 Alexa 488 and 20 Alexa 594 dyes were attached successfully, then all Alexa 488 and Alexa 594 dyes were attempted to be combined for the given frame conformation. If the dyes did not lead to steric clashes, then the distance between the dyes was saved. Transfer efficiencies per distance were determined using the Förster formula with *R*_0_ = 6 nm. For each frame, the mean transfer efficiency was calculated and used for the reweighting procedure.

The maximum entropy method COPER was utilized to reweight simulation ensembles to match experimental mean transfer efficiencies.^100^ Briefly, the experimental mean transfer efficiencies as well as their associated errors listed in Table S2 were used as inputs to generate weights per frame that yield a global solution satisfying the inputs. The generated weights were then used to extract quantify conformational properties from the simulated ensembles.

### Analyses of ABSINTH Simulations

All analyses were performed using the Python-based simulation analysis package SOURSOP.^101^ The weights extracted from COPER were used as inputs for the various analysis routines performed. Internal scaling profiles were calculated using the get_internal_scaling_RMS() analysis routine. The get_scaling_exponent() analysis routine was used to extract the best estimates of *A*_0_, the prefactor which reports on the chain persistence length, and scaling exponent, ν, from the standard homopolymer relationship, ⟨*R*_*i*,*j*_⟩ = ⟨⟨*r*_*i*,*j*_^2^⟩^1/2^⟩ = *A*_0_ |*i* – *j*|^ν^ for each simulated ensemble. The *A*_0_ and ν values extracted were then used as inputs into the analysis routine get_polymer_scaled_distance_map(). This routine determines how all residue distances compare to the best-fit standard homopolymer scaling behavior. The mode “scaled” was used which divides each weighted distance by the best-fit homopolymer model distance. Contact information was extracted using the analysis routine get_contact_map() with the mode “closest-heavy” and a contact distance threshold of 10 Å. The radius of gyration, asphericity, and secondary structure information per frame were calculated using the get_radius_of_gyration(), get_asphericity(), and get_secondary_structure_DSSP() analysis routines, respectively. Contact networks were generated using the Python package NetworkX. When contacts (nodes in Figure 3E and Figure S10) and distance averages (Figure S10) were extracted per residue (*i*), averages were taken only over residues greater than two residues away in linear sequence space, i.e., *j* > *i* + 2 and *j* < *i* – 2.

### Hydrophobicity Scale (HPS) Model Optimization and Simulations

We used the hydrophobicity scale model representation of disordered proteins,^63^ in which each residue is represented by a single bead with size based on average residue volumes in crystal structures, linked by harmonic bonds with equilibrium length 0.38 nm and spring constant 481.4 kJ nm^−2^ mol^−1^. Interactions of each bead are determined by a scalar parameter λ characterizing “stickiness” with other beads. The value λ was based on hydrophobicity scales in the original model but should not be literally interpreted as hydrophobicity. Pairwise interactions between the beads are described by a modified Weeks–Chandler–Anderson potential in which the attractive part is determined by the arithmetic mean of the λ-values of the two beads. Further details are as described by Dannenhoffer-Lafage and Best.^38^ The dyes were represented as shown in Figure S5, and force field parameters are given in Table S3. The dyes are linked by harmonic bonds with an equilibrium length of 0.38 nm and a spring constant of 481.4 kJ nm^−2^ mol^−1^. The shapes of the dyes are maintained by harmonic angle potential spring constants of 48.14 kJ rad^−2^ mol^−1^. The equilibrium angles for branch points were π/2 and π elsewhere. Note that harmonic potentials for bond angles were only applied to the colored beads in Figure S5 and not to the dye linker beads. The mass of each dye bead was set to 100 atomic mass units.

Langevin dynamics was propagated at a temperature of 300 K with a time step of 10 fs and a friction coefficient of 1.0 ps^–1^ using the LAMMPS package, with typical run lengths of 600 ns for each protein, discarding the first 100 ns for equilibration. Run input files and final parameters are available on Zenodo at 10.5281/zenodo.11397637. Force balance optimization was performed as described by Dannenhoffer-Lafage and Best,^38^ optimizing the FRET efficiency and using a learning rate of 5.0. The initial parameters for the protein were obtained from the M1 parameter set of Tesei et al.,^40^ while those for the dyes were obtained from a grid search over dye and linker λ values. Furthermore, L2 starting point regularization was employed with initial parameters used as the starting point and a regularization strength of 0.0001. Simulations were performed at an ionic strength of 185 mM, with CF660R or Alexa 594 attached to Cys5 and Cy3B or Alexa 488 attached to Cys64. Permuting the dyes led to an average difference in ⟨*E*⟩ of 0.018, which was thus not taken into account in our comparison. Benchmark simulations of the CALVADOS data set^40^ were performed with the CALVADOS M1 parameters and with our optimized “Linker-HPS” parameters (Figure S11).

Simulations of PNt, ibb, n49, nul, and nus were propagated at 298 K with a time step of 10 fs, a friction coefficient of 1.0 ps^–1^, and a Debye length corresponding to an ionic strength of 190 mM for PNt and 165 mM for ibb, n49, nul, and nus. PNt was simulated for 60 μs, and the first 10 μs was discarded for equilibration. The simulations of ibb, n49, nul, and nus were propagated for 10 μs, and the first microsecond was discarded for equilibration.

## Supporting Information Available

Selection scheme, disorder scores, and circular dichroism spectra for the 16 IDR sequences, single-molecule analysis of sκh labeled with Cy3B/CF660R, coarse-grained dye structures, SAXS and NMR data, correlations between different sequence parameters and average transfer efficiency, and additional ABSINTH and HPS model analyses (Figures S1−S13); summary of UniProt11 identifiers and important physicochemical parameters of the IDRs, original (CALVADOS 112 M3), optimized, and CALVADOS 213 short-range interaction parameters (λ) (Tables S1−S3) (PDF)
