# Supplementary material for: Identifying Sequence Effects on Chain Dimensions of Disordered Proteins by Integrating Experiments and Simulations
Source: JACS Au. 2024 Nov 14;4(12):4729–43. doi: 10.1021/jacsau.4c00673 (PMC11672150; doi:10.1021/jacsau.4c00673)
Supplement: Supplementary file 1 — au4c00673_si_001.pdf [file au4c00673_si_001.pdf]

Supporting Information

Identifying sequence effects on chain dimensions of disordered proteins by integrating experiments and simulations

Andrea Holla[1], Erik W. Martin[2], Thomas Dannenhoffer-Lafage[3], Kiersten M. Ruff[4], Sebastian L. B. König[1,5], Mark F. Nüesch[1], Aritra Chowdhury[1], John M. Louis[3], Andrea Soranno[1,6], Daniel Nettels[1], Rohit V. Pappu[4]*, Robert B. Best[3]*, Tanja Mittag[2]*, Benjamin Schuler[1,7]*

[1]Department of Biochemistry, University of Zurich, Winterthurerstrasse 190, 8057 Zurich, Switzerland
[2]Department of Structural Biology, St. Jude Children's Research Hospital, 262 Danny Thomas Place, Memphis, TN 38105, USA
[3]Laboratory of Chemical Physics, National Institute of Diabetes and Digestive and Kidney Diseases, National Institutes of Health, Bethesda, MD 20892-0520, USA
[4]Department of Biomedical Engineering and Center for Biomolecular Condensates, Washington University in St. Louis, St. Louis, MO 63130, USA
[5]Present Address: Federal Food Safety and Veterinary Office, 3003 Bern, Switzerland
[6]Department of Biochemistry and Molecular Biophysics, Center for Biomolecular Condensates, Washington University in St. Louis, St. Louis, USA
[7]Department of Physics, University of Zurich, Winterthurerstrasse 190, 8057 Zurich, Switzerland

*Address correspondence to Rohit V. Pappu (pappu@wustl.edu), Robert B. Best (robert.best2@nih.gov), Tanja Mittag (Tanja.Mittag@stjude.org), or Benjamin Schuler (schuler@bioc.uzh.ch)

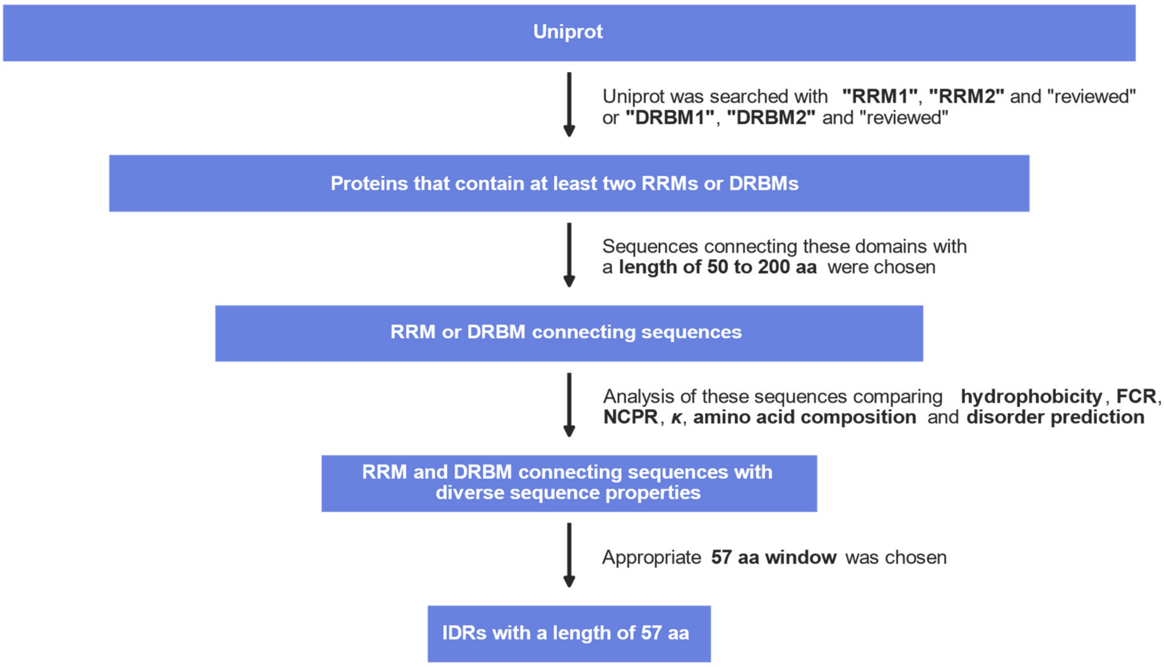

Figure S1. Selection scheme for the 16 IDR sequences.

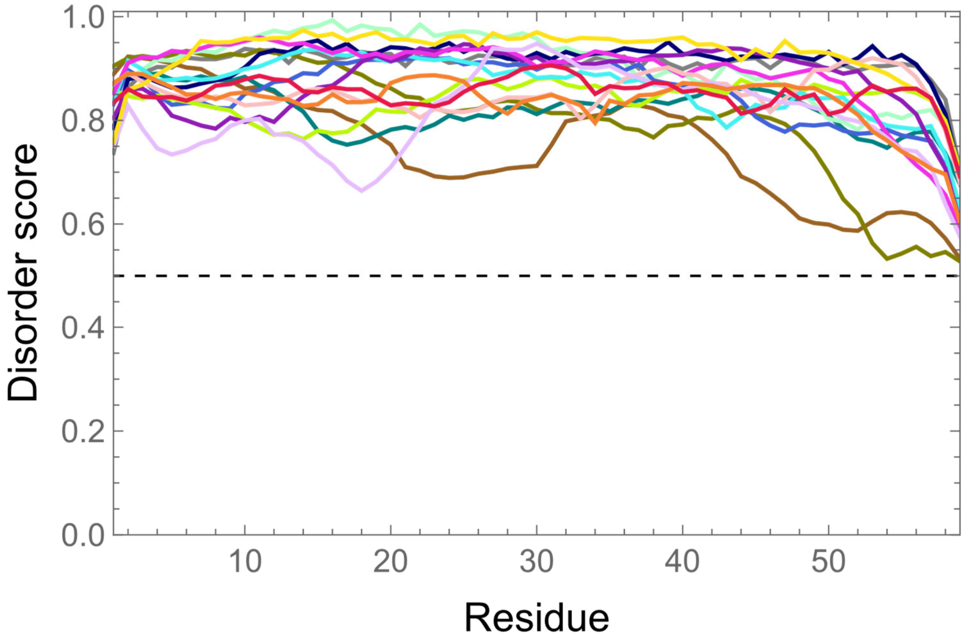

Figure S2. Metapredict[1] disorder score for the 16 IDR sequences. The disorder score is >0.5 (dashed line) for all sequences. Color code as in Fig. 1.

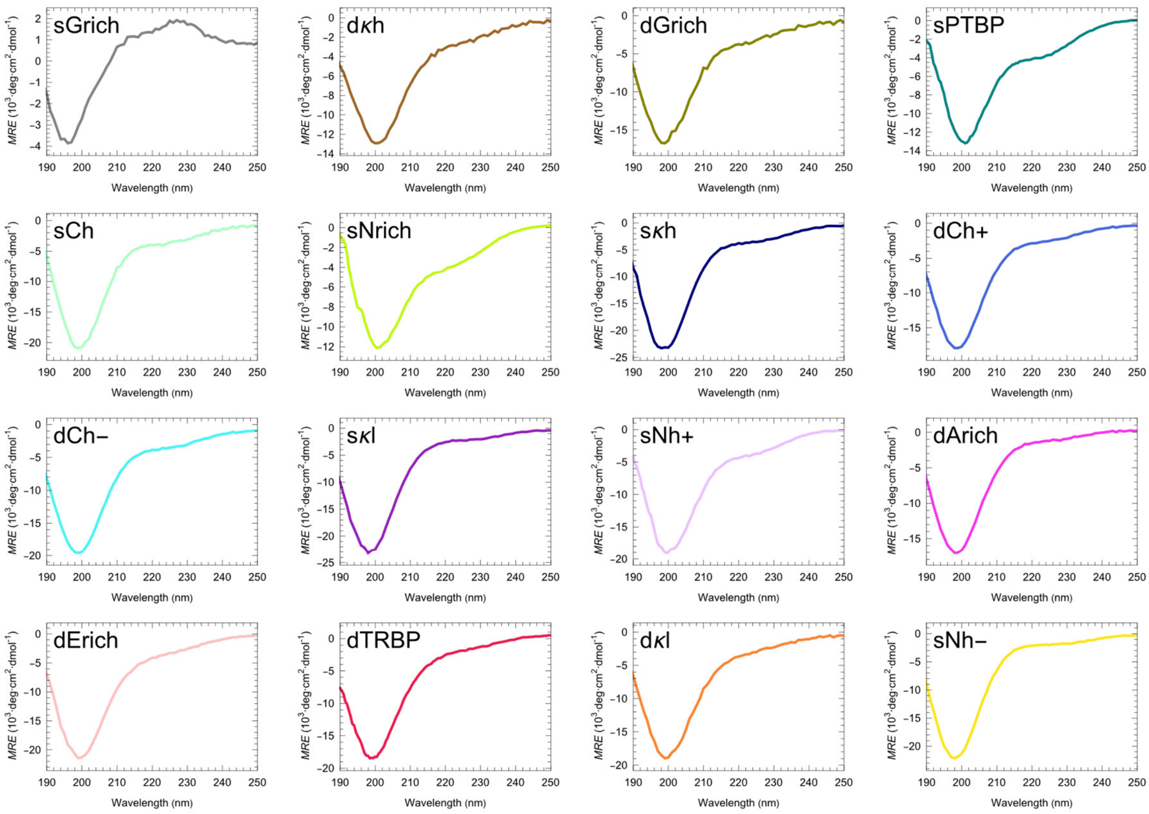

Figure S3. Circular dichroism spectra of the IDRs indicate the absence of pronounced secondary structure. The detectable sequence-specific differences between spectra are difficult to quantify reliably owing to the uncertainty in protein concentration measurements in sequences lacking aromatic amino acids (see Methods).

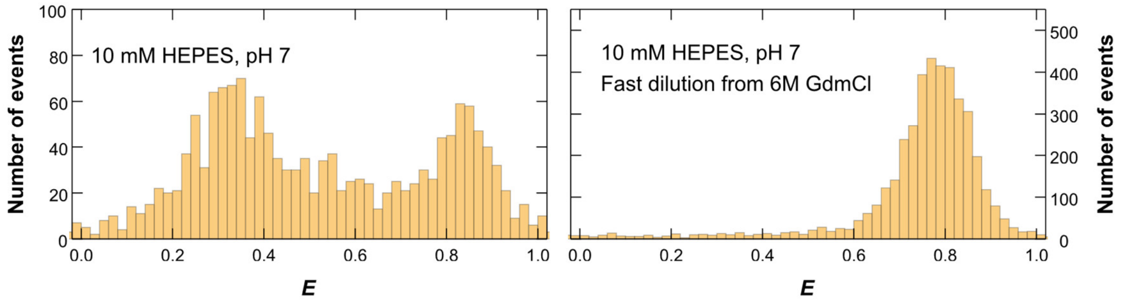

Figure S4. Single-molecule analysis of sκh labeled with Cy3B/CF660R identifies misfolding. Initial analysis of sκh in 10 mM 2-[4-(2-hydroxyethyl)piperazin-1-yl]ethane-1-sulfonic acid (HEPES), pH 7 (left histogram) revealed the presence of misfolded or aggregated species in addition to the monomeric form. However, by rapidly diluting sκh to picomolar concentrations from the fully denatured state in 6 M guanidine chloride (GdmCl), the formation of these undesirable species could be prevented (right histogram). Single-molecule spectroscopy thus helps to identify issues with misfolding or aggregation that can then be eliminated by a suitable choice of solution conditions and the exceedingly low protein concentrations used.

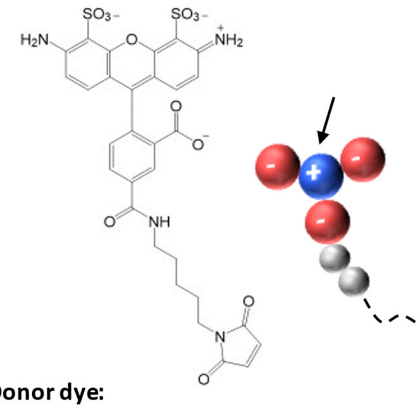

Donor dye:
Alexa Fluor 488 (isoform)

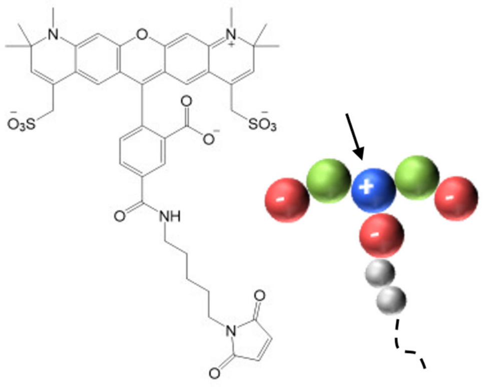

Acceptor dye:
Alexa Fluor 594 (isoform)

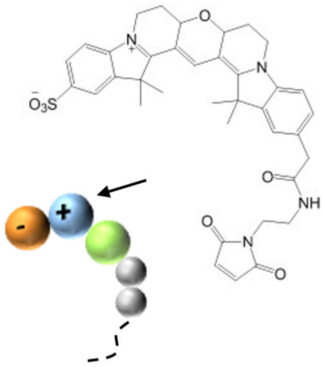

Donor dye: Cy3B

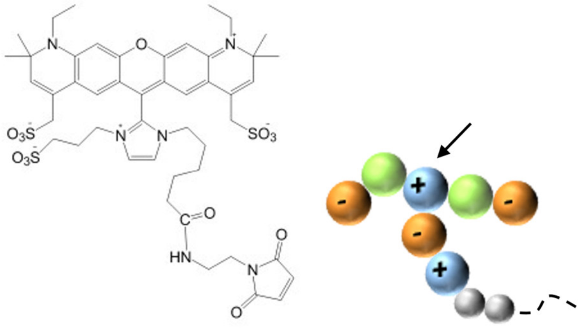

Acceptor dye: CF660R

Figure S5. Chemical structures of dyes Alexa 488/594 and Cy3B/CF660R, and their representation using beads for HPS model. For both the Alexa 488/594 and Cy3B/CF660R dye pairs, we employ a representation with neutral, positively charged, and negatively charged beads. In the case of Alexa 488/594, these beads are color-coded as green (neutral), blue (positive), and red (negative), respectively; for Cy3B/CF660R, the corresponding beads are light green (neutral), light blue (positive) and orange (negative), with an arrow indicating the bead used for quantifying dye-dye distances. Additionally, dye linker beads (gray) are included in the representation.

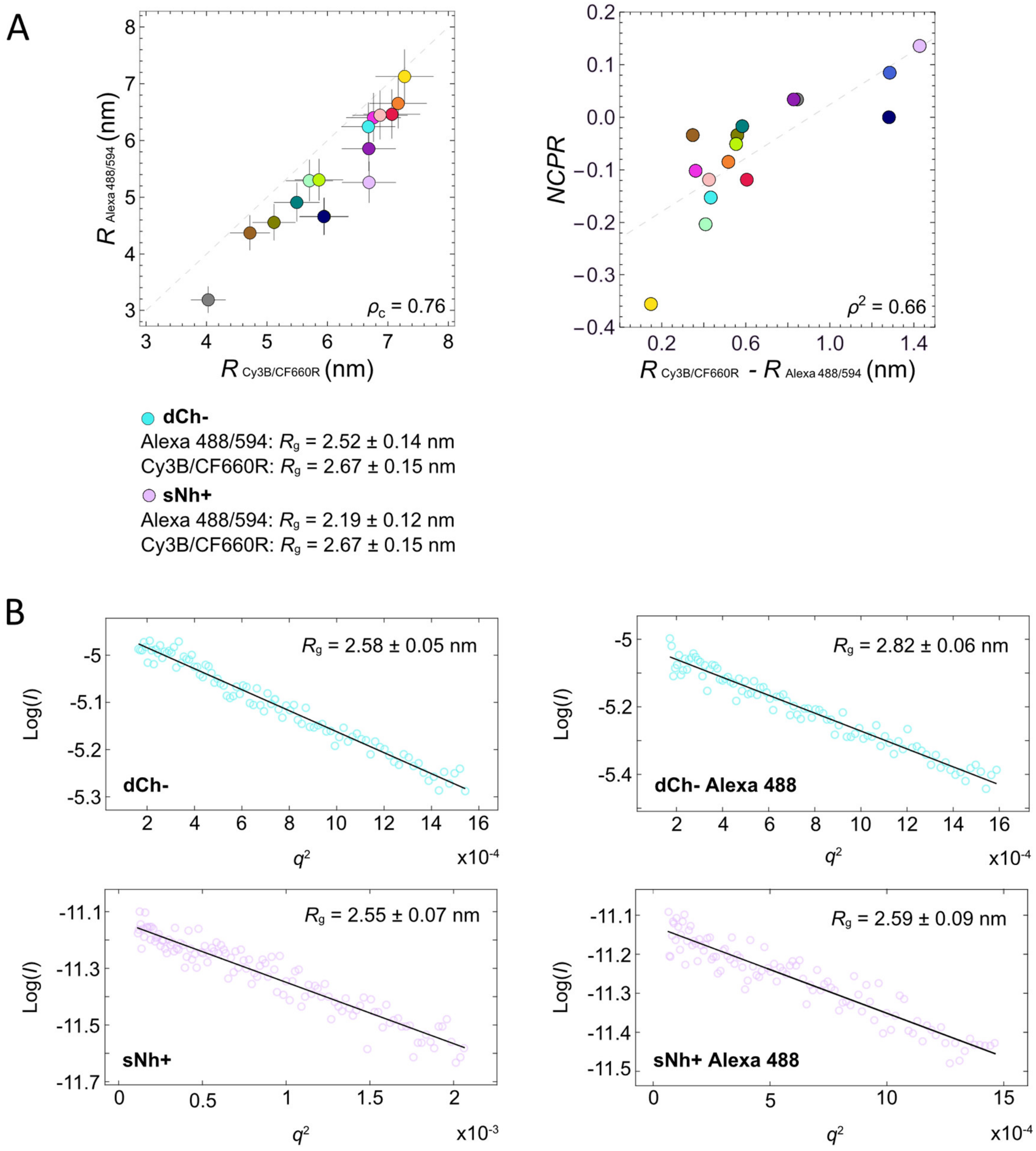

Figure S6. Comparison of the IDRs labeled with the dye pairs Alexa 488/594 and Cy3B/CF660R by FRET, SAXS, and NMR. (A) Correlation between the root-mean squared distance (*R*) of the IDRs labeled with Alexa 488/594 and Cy3B/CF660R, respectively, from single-molecule FRET. *R* was inferred from the mean transfer efficiency assuming a SAW-*ν* distance distribution[2], with error bars based on a systematic uncertainty of ±7% in the Förster radius[3] used to calculate *R*; radii of gyration (R_g) for comparison with (B) were estimated from *R* and *ν*.[2] Color code for the sequences as in Fig. 1. (B) SAXS measurements of dCh- and sNh+ unlabeled (left) and double-labeled with Alexa 488 (right). R_g was obtained from Guinier fits to the linear region of the scattering curve. [continued on the next page]

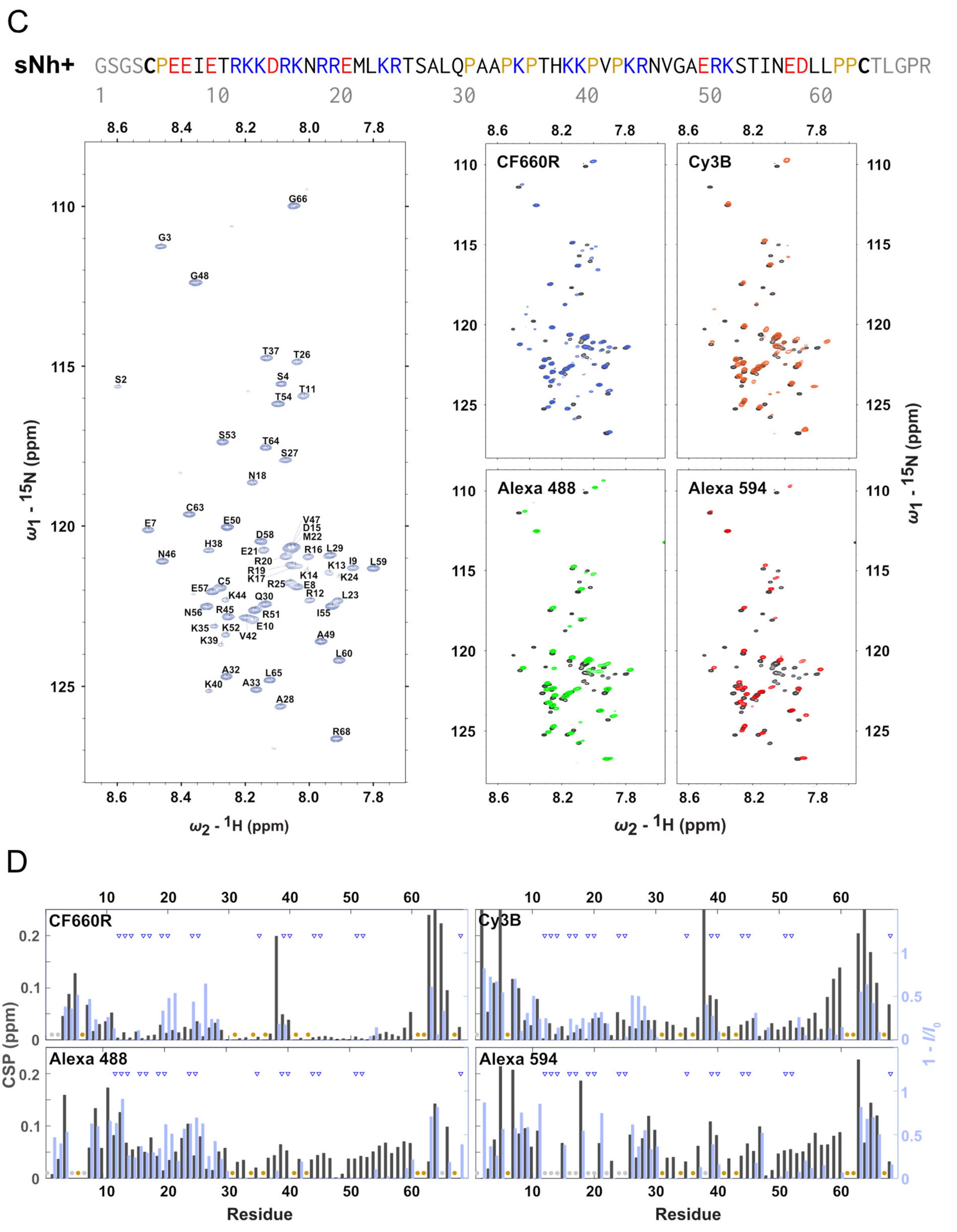

Figure S6 [continued from previous page]. Comparison of the IDRs labeled with the dye pairs Alexa 488/594 and Cy3B/CF660R by FRET, SAXS, and NMR. (C) Left: Assigned HSQC spectrum of unlabeled sNh+; right: HSQC spectra of sNh+ double-labeled with the fluorophores indicated (colored spectra) and of the unlabeled IDRs (black spectra). (D) Chemical shift perturbations (CSP, dark gray bars) and resonance intensity decrease (light blue bars) upon double-labeling of sNh+ with the fluorophores indicated; blue triangles: positively charged amino acids; brown circles: resonances that were assigned in the unlabeled peptide but could not be assigned in the labeled peptide; light gray circles: proline residues.

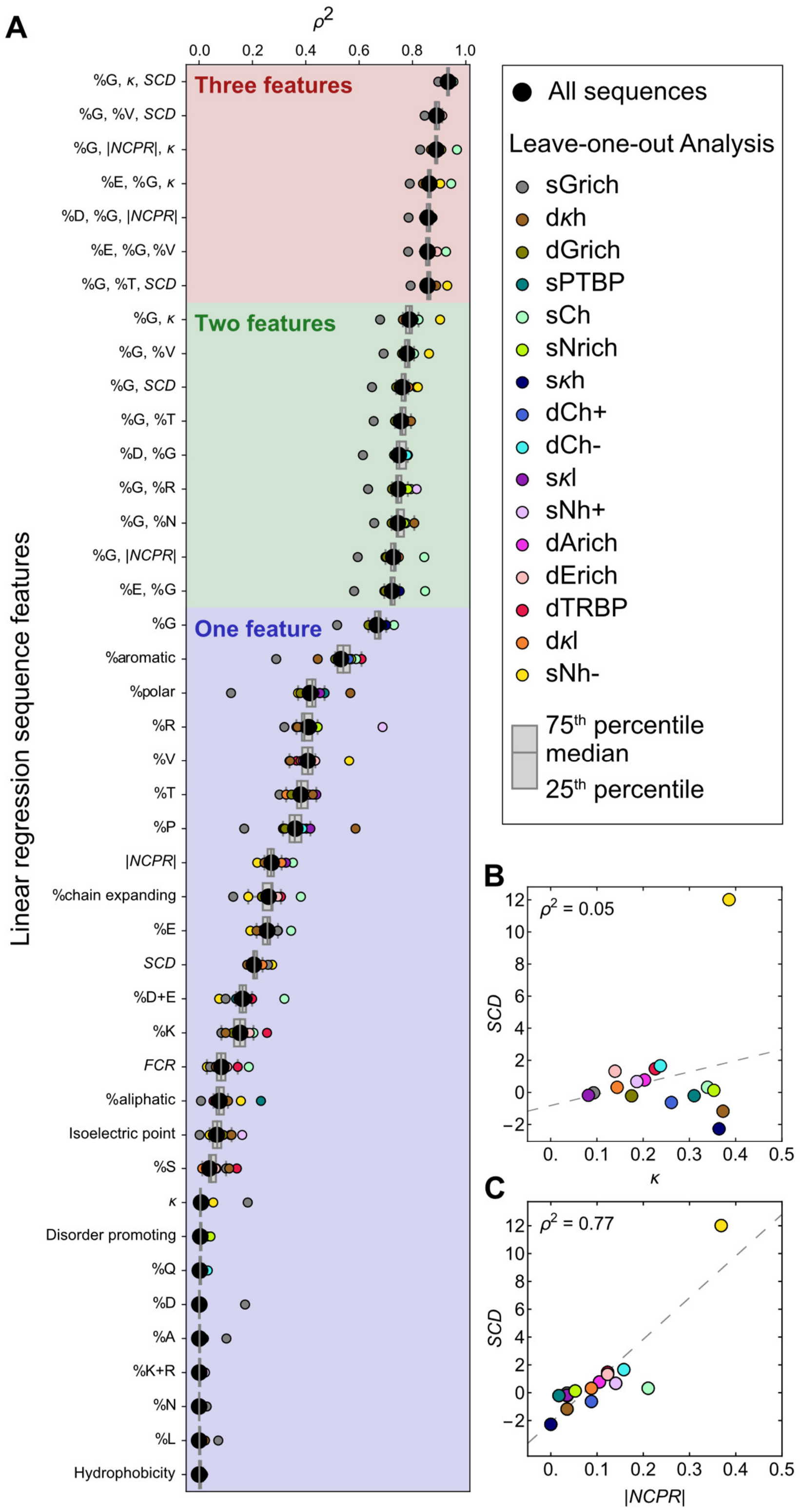

Figure S7. Correlations between different sequence parameters and average transfer efficiency. (A) Single-letter codes for amino acids are used; aro: aromatic residues; *SCD*: sequence charge decoration. Color code for the sequences as in Fig. 1. ρ^2 analysis for linear regression of various compositional sequence features when including all sequences (black circle) or all sequences but one (colored circles). (B) and(C) Correlation analysis of κ and *SCD* and *SCD* and |*NCPR*| showing correlation between *SCD* and |*NCPR*|.

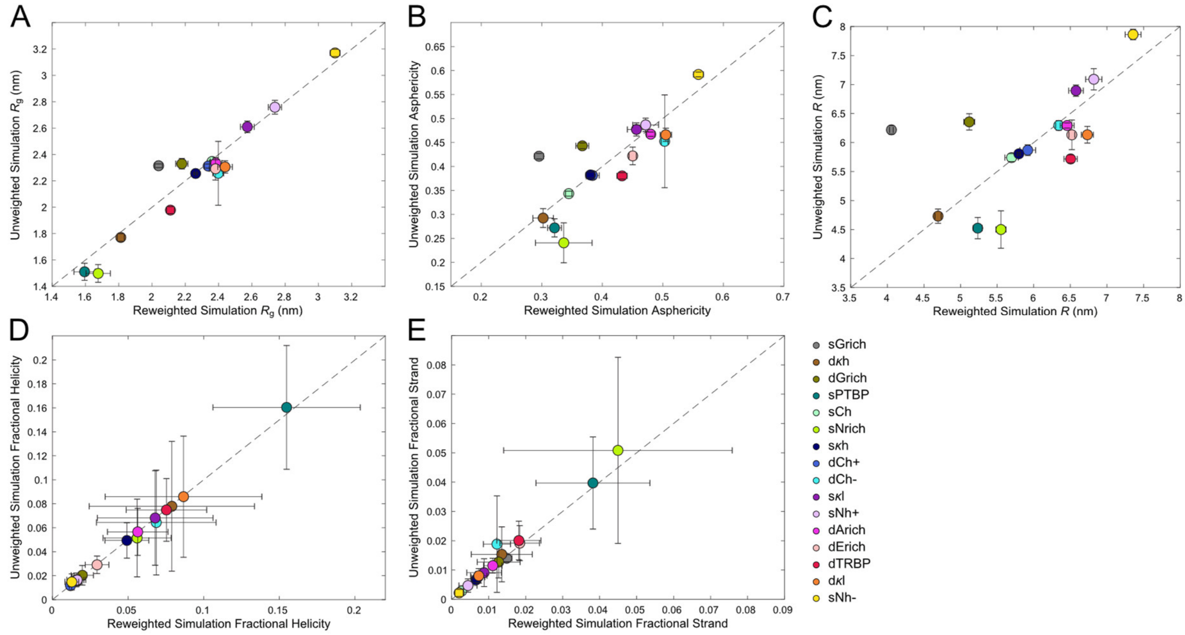

Figure S8. Comparisons of conformational parameters of unweighted (prior) and reweighted (posterior) ABSINTH ensembles. (A) The prior (ordinate) vs posterior values (abscissa) for (A) radius of gyration, R_g, (B) asphericity, (C) root-mean-squared end-to-end distance, R, (D) DSSP[4] fractional helical content, and (E) DSSP fractional strand content. For asphericity, values between 0.1 and 0.3 refer to roughly spherical envelopes for the ensembles. Values greater than 0.5 refer to prolate ellipsoids[5]. In all panels, the dashed lines define the lines of equality between prior and posterior values. Error bars represent the standard errors in the estimates of the mean values, which are shown as symbols.

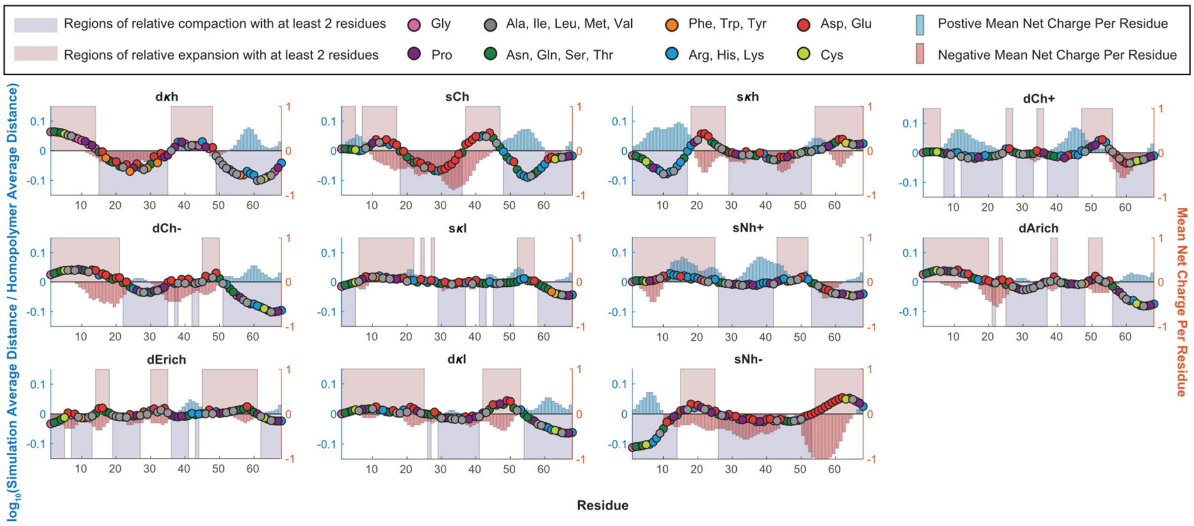

Figure S9. Degree of local expansion and contraction relative to the best fit homopolymer model. Circles are colored by their residue type. Consecutive regions of at least two residues that show compaction or expansion relative to the homopolymer model are shown by blue and red boxes, respectively. Mean net charge per residue profiles, averaged over five residue stretches, are shown as bar plots. Only the results for the sequences for which reweighting resulted in a Kullback-Leibler divergence below 0.1 are shown.

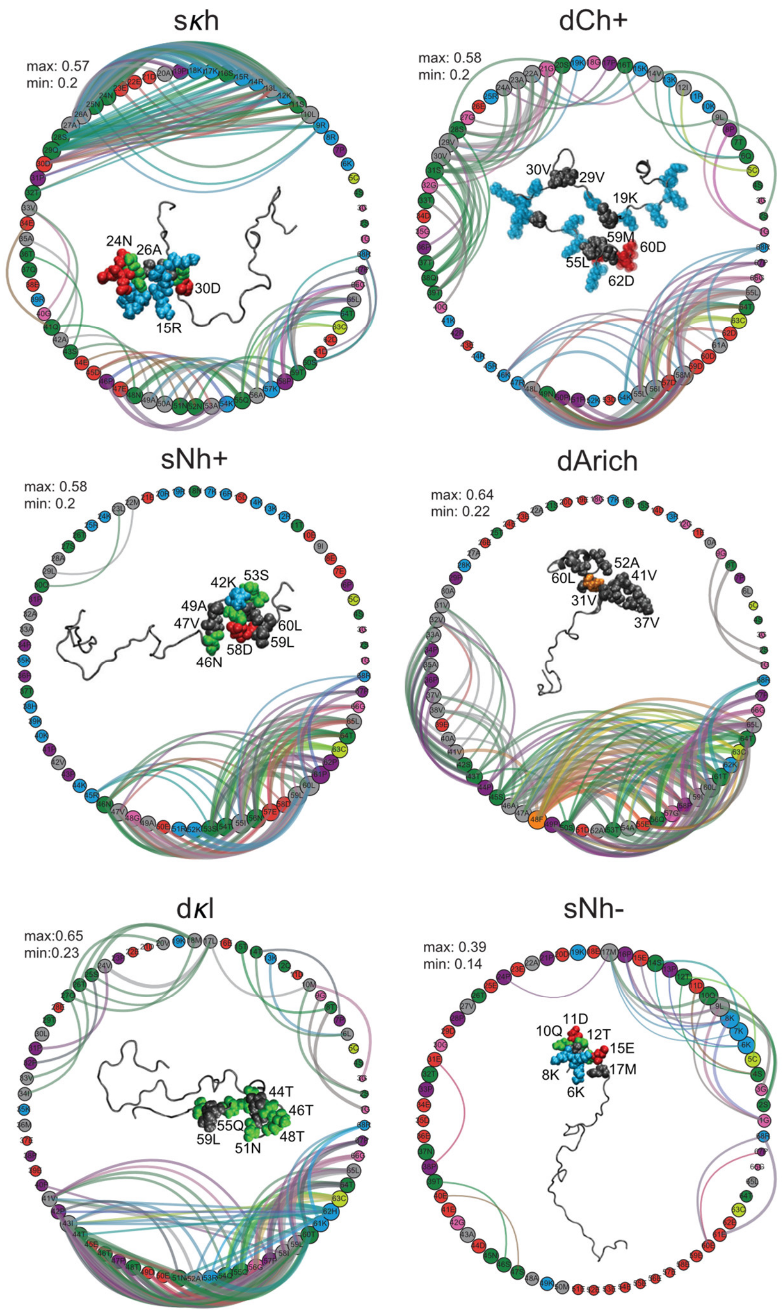

Figure S10. Contact networks for the IDRs not shown in Fig. 3E. Residues are shown as nodes with the circle size correlated to the mean contact probability between that residue and all other residues greater than two residues away in linear sequence space. Edges are drawn between two residues if they are at least 35 % of the maximum contact probability observed for that IDR. The maximum and minimum contact probabilities used to draw edges are listed in the figure as min and max. The width of the edge is 10 times the mean contact probability. Here, a contact distance of 10 Å is used such that charge interactions can be observed. Gly is shown in pink, Ser, Thr, Asn, and Gln in green, Arg, Lys, and His in blue, Asp and Glu in red, Phe, Trp, and Tyr in orange, Met, Val, Ile, Leu, and Ala in grey, Pro in purple, and Cys in lime green. Edge colors are the mixture of the interacting residue colors. Representative snapshots are visualized using VMD[6] and chosen by finding the frame that has the highest weight with a radius of gyration (R_g) within 0.5 Å of the average R_g for the IDR. Only the results for the sequences for which reweighting resulted in a Kullback-Leibler divergence below 0.1 are shown.

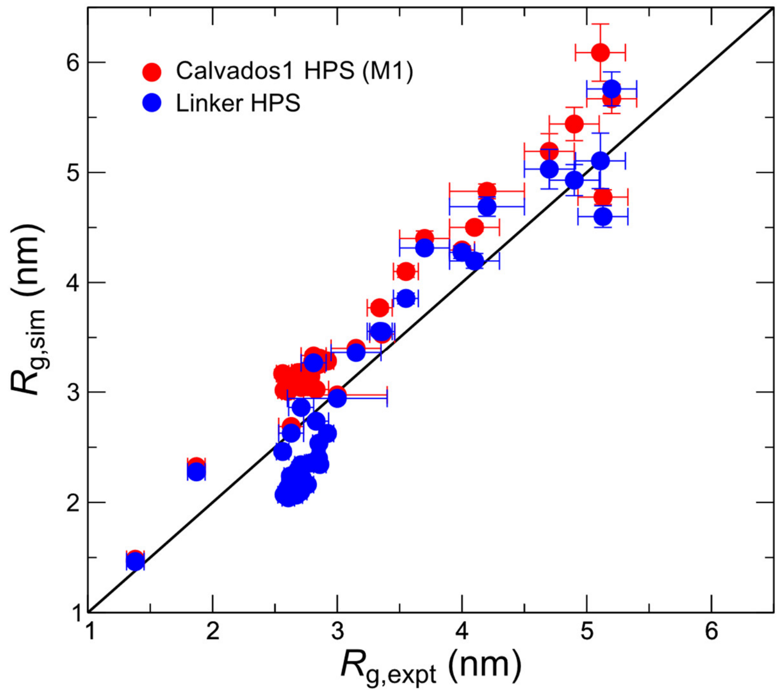

Figure S11. Comparison of CALVADOS 1 HPS model[7] (M1) and "Linker HPS" model optimized here applied to the CALVADOS 1 training set. The radii of gyration, $R_{g,sim}$, were computed from simulations of the 42 proteins in the CALVADOS training set[7] using the CALVADOS 1 (M1) λ values (red) or the λ values we are presenting here ("Linker HPS", blue), and compared with experimental radii of gyration, $R_{g,expt}$. The cluster of A1 LCD-related sequences is located roughly between 2.5 and 3.0 nm in $R_{g,expt}$.

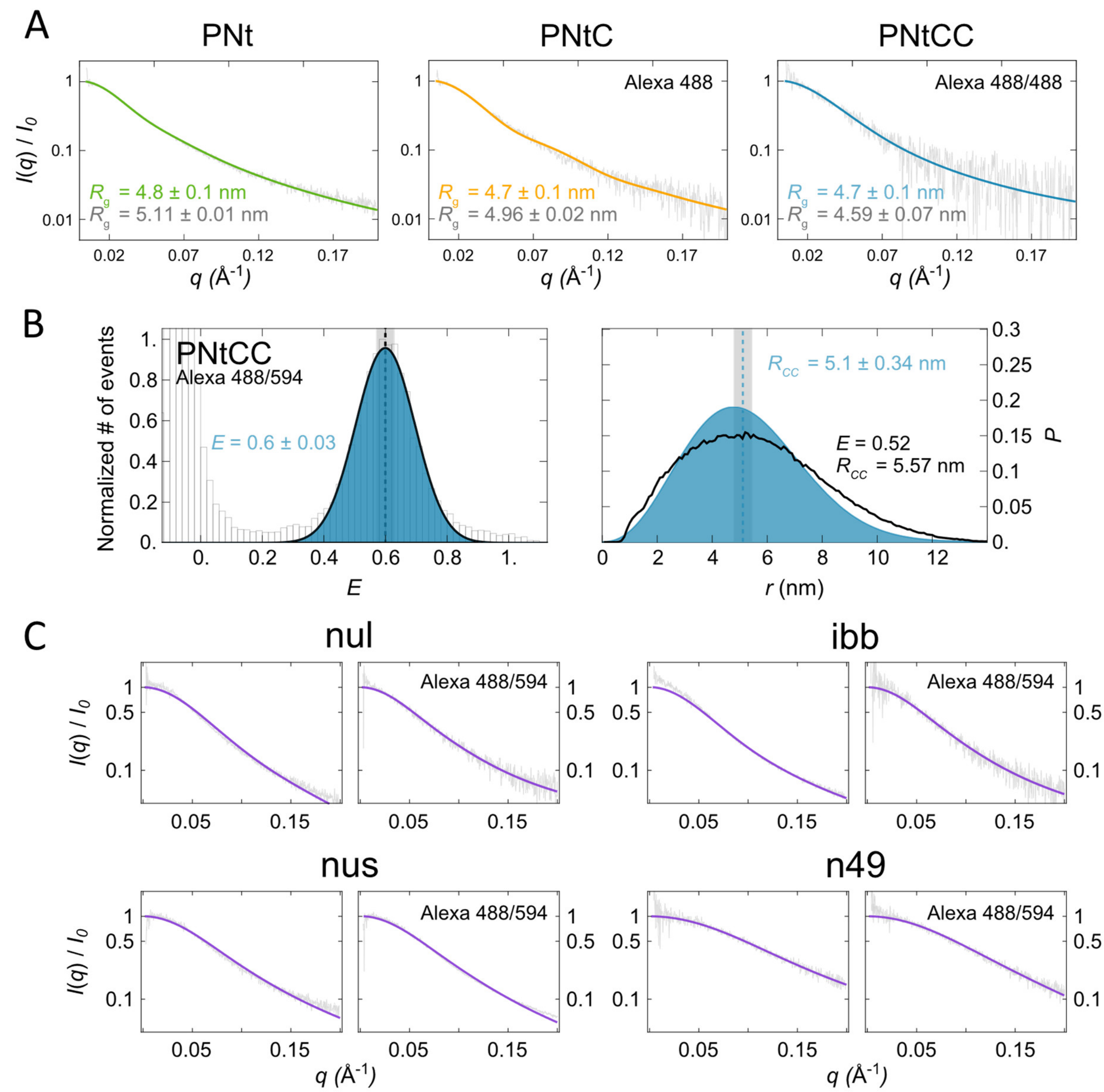

Figure S12. (A) SAXS curves of PNt variants[8], left to right: unlabeled (PNt), labeled with one Alexa 488 dye (PNtC), and labeled with Alexa 488 at both Cys residues (PNtCC), compared with results based on the simulations with the optimized HPS model (colored lines). (B) Transfer efficiency histogram of PNtCC labeled with Alexa 488/594 measured in 10 mM Tris and 175 mM KCl, pH 7.4 (ionic strength 188 mM, accounting for residual GdmCl from dilution of labeled protein), fit with a Gaussian peak function (blue) to estimate the mean transfer efficiency. Distance distributions, $P(r)$, based on the SAW-ν model[2] (blue) and the optimized coarse-grained HPS model (black line). Vertical dashed line indicates the root-mean squared distance (R_{cc}) from SAW-ν, with a gray error band based on a systematic uncertainty of ±7% in the Förster radius[9]. The difference between experimental and simulation FRET efficiencies is likely due to the limitations in the HPS model in reproducing the sequence-specific dimensions of the 88-residue segment between the labels; the discrepancy is within the range of deviations obtained for the linker IDRs (Fig. 4B). Note that the R_g estimated from experimental or simulated FRET using the SAW-ν and extrapolated to the full sequence length is 4.51 and 5.07 nm respectively, lying on either side of the estimate from SAXS. (C) Comparison of experimental SAXS curves of the disordered proteins nul, ibb, nus and n49, unlabeled and labeled with Alexa 488/594 (gray lines) from Fuertes *et al.*[10] with SAXS curves calculated from simulations with the optimized HPS model including dyes (violet lines).

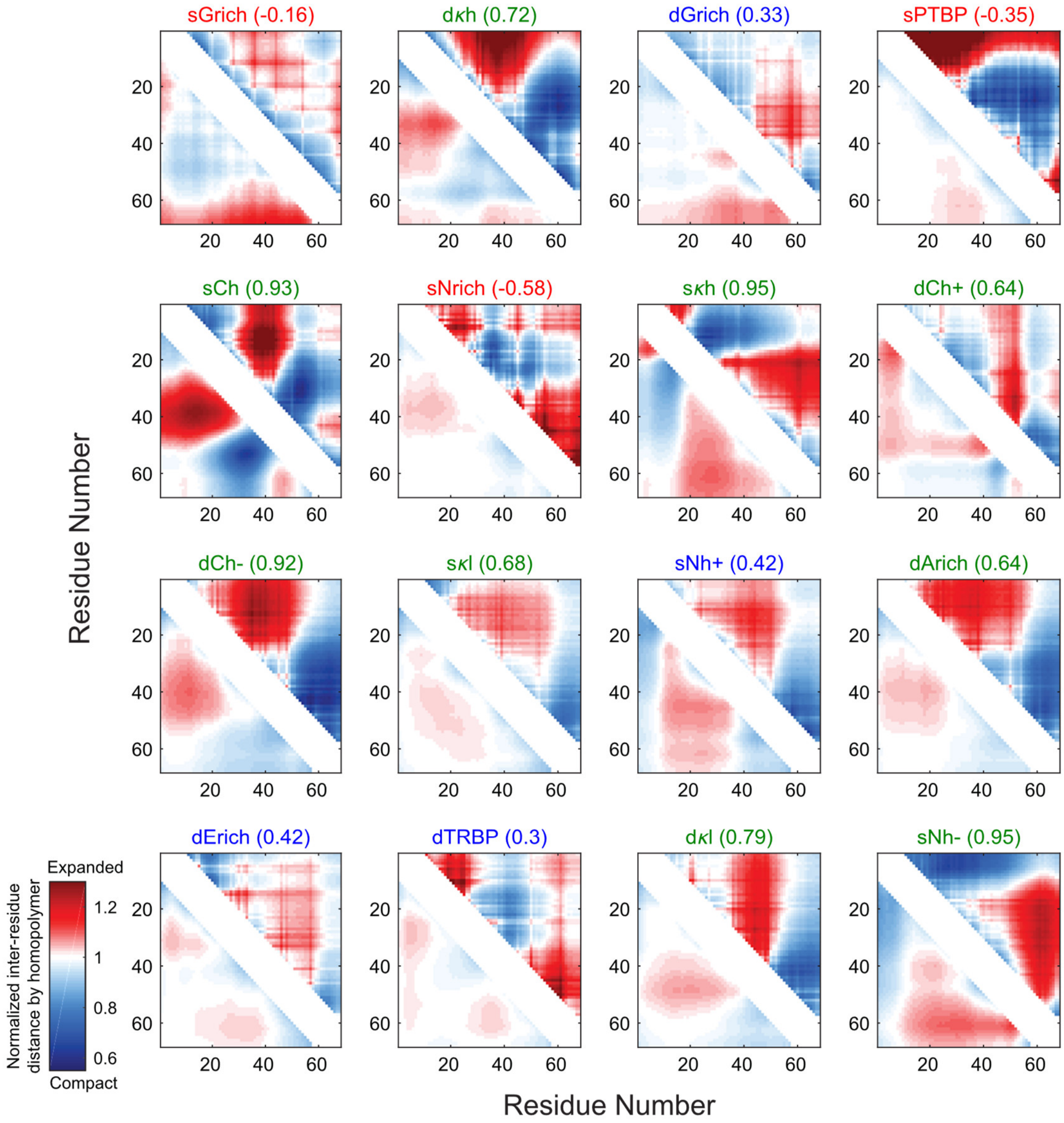

Figure S13. Comparison of normalized intra-chain distances computed using the reweighted ABSINTH simulations (upper triangle) and the HPS simulations with Alexa 488/594 (lower triangle). Results are shown with a minimum spacing of 10 amino acids and relative to the value from the best fit of a homopolymer model (see Fig. 3). Regions of local expansion relative to the equivalent homopolymer are shown in red, areas of local compaction in blue (see color scale). The title for each panel includes the name of the sequence, and the numbers in parentheses are Pearson correlation coefficients between $O(10^3)$ pairs of normalized distances from the two models. Strong positive correlations corresponding to correlation coefficients greater than +0.6 are marked in green. Sequences marked in blue show weak positive correlations between +0.3 and +0.5. For three sequences, the correlation coefficients are negative, implying that the reweighted ABSINTH ensembles are inconsistent the HPS ensembles. For sGrich, the HPS ensemble shows uniform compaction through the middle of the chain with the ends avoiding one another. In contrast, the reweighted ABSINTH ensemble yields numerous local loops and very few long-range attractions. For sNrich, the regions of attractions and repulsions are inverted across the two models. Finally, for sPTBP, the reweighted ABSINTH ensemble shows some helicity and a preference for turn-like structures. The HPS ensemble shows weaker overall contact preferences, suggestive of interactions closer to the homopolymer model than those in ABSINTH.

	UniProt	Netcharge	*FCR*	|*NCPR*|	Hydrophobicity	*κ*	*SCD*
sNh−	Q9Y4C8	−21	0.54	0.37	0.26	0.39	12.01
dκl	Q6NXA4	−5	0.26	0.09	0.41	0.14	0.32
dTRBP	Q15633	−7	0.16	0.12	0.49	0.23	1.47
dErich	Q6GPZ1	−7	0.23	0.12	0.44	0.14	1.32
dArich	Q12906	−6	0.25	0.11	0.46	0.20	0.77
sNh+	O74968	+8	0.42	0.14	0.33	0.19	0.67
sκl	Q54PB2	+2	0.35	0.04	0.36	0.08	−0.18
dCh−	Q91550	−9	0.30	0.16	0.39	0.24	1.66
dCh+	P25159	+5	0.37	0.09	0.37	0.26	−0.63
sκh	A7TQR2	0	0.39	0.00	0.30	0.36	−2.27
sNrich	P32831	−3	0.12	0.05	0.43	0.35	0.13
sCh	P37838	−12	0.60	0.21	0.19	0.34	0.32
sPTBP	P26599	−1	0.09	0.02	0.55	0.31	−0.21
dGrich	P24785	−2	0.25	0.04	0.38	0.17	−0.22
dκh	Q9NS39	−2	0.21	0.04	0.45	0.37	−1.17
sGrich	Q43349	+2	0.18	0.04	0.35	0.09	−0.03

Table S1: Summary of UniProt[11] identifiers and important physicochemical parameters of the IDRs

	$\overline{\langle E \rangle}_{A488A594}$	$\overline{\langle E \rangle}_{Cy3bCF660R}$	$R_{A488A594}$ [nm]	$R_{Cy3bCF660R}$ [nm]	$\nu_{A488A594}$	$\nu_{Cy3bCF660R}$
sNh−	0.36 ± 0.02	0.42 ± 0.03	$7.1^{+0.5}_{-0.5}$	$7.3^{+0.5}_{-0.5}$	$0.61^{+0.02}_{-0.02}$	$0.61^{+0.02}_{-0.02}$
dκl	0.41 ± 0.02	0.43 ± 0.03	$6.7^{+0.4}_{-0.4}$	$7.2^{+0.5}_{-0.5}$	$0.59^{+0.02}_{-0.02}$	$0.61^{+0.02}_{-0.02}$
dTRBP	0.437 ± 0.006	0.45 ± 0.03	$6.5^{+0.4}_{-0.4}$	$7.1^{+0.5}_{-0.5}$	$0.59^{+0.02}_{-0.02}$	$0.61^{+0.02}_{-0.02}$
dErich	0.44 ± 0.03	0.47 ± 0.03	$6.4^{+0.4}_{-0.4}$	$6.9^{+0.5}_{-0.5}$	$0.59^{+0.02}_{-0.02}$	$0.60^{+0.02}_{-0.02}$
dArich	0.445 ± 0.009	0.48 ± 0.02	$6.4^{+0.4}_{-0.4}$	$6.8^{+0.4}_{-0.4}$	$0.58^{+0.02}_{-0.02}$	$0.60^{+0.02}_{-0.02}$
sNh+	0.610 ± 0.007	0.49 ± 0.03	$5.3^{+0.4}_{-0.4}$	$6.7^{+0.4}_{-0.4}$	$0.54^{+0.02}_{-0.02}$	$0.59^{+0.02}_{-0.02}$
sκl	0.52 ± 0.02	0.49 ± 0.03	$5.9^{+0.4}_{-0.4}$	$6.7^{+0.4}_{-0.4}$	$0.56^{+0.02}_{-0.02}$	$0.59^{+0.02}_{-0.02}$
dCh−	0.46 ± 0.03	0.50 ± 0.03	$6.2^{+0.4}_{-0.4}$	$6.7^{+0.4}_{-0.4}$	$0.58^{+0.02}_{-0.02}$	$0.59^{+0.02}_{-0.02}$
dCh+	0.70 ± 0.02	0.59 ± 0.03	$4.7^{+0.3}_{-0.3}$	$5.9^{+0.4}_{-0.4}$	$0.51^{+0.02}_{-0.02}$	$0.57^{+0.02}_{-0.02}$
sκh	0.706 ± 0.002	0.595 ± 0.005	$4.7^{+0.3}_{-0.3}$	$5.9^{+0.4}_{-0.4}$	$0.51^{+0.02}_{-0.02}$	$0.57^{+0.02}_{-0.02}$
sNrich	0.60 ± 0.02	0.61 ± 0.02	$5.3^{+0.4}_{-0.4}$	$5.9^{+0.4}_{-0.4}$	$0.54^{+0.02}_{-0.02}$	$0.56^{+0.02}_{-0.02}$
sCh	0.605 ± 0.003	0.63 ± 0.04	$5.3^{+0.4}_{-0.4}$	$5.7^{+0.4}_{-0.4}$	$0.54^{+0.02}_{-0.02}$	$0.56^{+0.02}_{-0.02}$
sPTBP	0.666 ± 0.006	0.66 ± 0.02	$4.9^{+0.3}_{-0.3}$	$5.5^{+0.4}_{-0.4}$	$0.52^{+0.02}_{-0.02}$	$0.55^{+0.02}_{-0.02}$
dGrich	0.72 ± 0.02	0.71 ± 0.02	$4.6^{+0.3}_{-0.3}$	$5.1^{+0.4}_{-0.4}$	$0.50^{+0.02}_{-0.02}$	$0.53^{+0.02}_{-0.02}$
dκh	0.75 ± 0.02	0.77 ± 0.02	$4.4^{+0.3}_{-0.3}$	$4.7^{+0.3}_{-0.3}$	$0.49^{+0.02}_{-0.02}$	$0.51^{+0.02}_{-0.02}$
sGrich	0.912 ± 0.004	0.861 ± 0.008	$3.2^{+0.2}_{-0.2}$	$4.0^{+0.3}_{-0.3}$	$0.42^{+0.02}_{-0.02}$	$0.47^{+0.02}_{-0.02}$

Table S2: Mean transfer efficiencies, $\langle E \rangle$, averaged from at least three independent measurements, the corresponding average root-mean squared distances, *R,* and scaling exponents, *ν,* from SAW-*ν*. The uncertainties for *R* and *ν* are based on a systematic uncertainty of ±7% in the Förster radius[9].

	Initial	Optimized	Calvados2
Ala	0.003	0.238	0.274
Arg	0.723	0.79	0.731
Asn	0.16	0.298	0.426
Asp	0.002	0.223	0.042
Cys	0.4	0.414	0.562
Gln	0.468	0.502	0.393
Glu	0.022	0.05	0.001
Gly	0.784	1.268	0.706
His	0.487	0.497	0.466
Ile	0.687	0.669	0.542
Leu	0.335	0.394	0.644
Lys	0.095	0.083	0.179
Met	0.993	0.982	0.531
Phe	0.871	0.915	0.867
Pro	0.471	0.299	0.359
Ser	0.487	0.572	0.463
Thr	0.274	0.181	0.371
Trp	0.753	0.753	0.989
Tyr	0.984	1.024	0.977
Val	0.428	0.267	0.208
Aneg	1	1.109	
Aneu	1	1.083	
Apos	1	1.089	
Cneg	0.5	0.424	
Cneu	0.5	0.432	
Cpos	0.5	0.452	
Lin	0.75	0.783	

Table S3. Original (CALVADOS 1[12] M3), optimized, and CALVADOS 2[13] short-range interaction parameters (λ). Aneg, Aneu, and Apos are the parameters for the negatively charged, neutral, and positively charged Alexa 488/594 dye beads, respectively. Cneg, Cneu, and Cpos are the parameters for the negatively charged, neutral, and positively charged Cy3B/CF660R dye beads, and Lin for the linking dye beads, respectively (see Fig. S5). Values for the parameters σ and ε in the HPS model were taken from the previous version of the model[14]; for each dye bead, a value of 0.582 nm was used for σ.

References

1. Emenecker, R. J.; Griffith, D.; Holehouse, A. S., Metapredict: a fast, accurate, and easy-to-use predictor of consensus disorder and structure. *Biophys. J.* **2021,** *120* (20), 4312-4319.
2. Zheng, W.; Zerze, G. H.; Borgia, A.; Mittal, J.; Schuler, B.; Best, R. B., Inferring properties of disordered chains from FRET transfer efficiencies. *J. Chem. Phys.* **2018,** *148* (12), 123329.
3. Hellenkamp, B.; Schmid, S.; Doroshenko, O.; Opanasyuk, O.; Kuhnemuth, R.; Rezaei Adariani, S.; Ambrose, B.; Aznauryan, M.; Barth, A.; Birkedal, V.; Bowen, M. E.; Chen, H.; Cordes, T.; Eilert, T.; Fijen, C.; Gebhardt, C.; Gotz, M.; Gouridis, G.; Gratton, E.; Ha, T.; Hao, P.; Hanke, C. A.; Hartmann, A.; Hendrix, J.; Hildebrandt, L. L.; Hirschfeld, V.; Hohlbein, J.; Hua, B.; Hubner, C. G.; Kallis, E.; Kapanidis, A. N.; Kim, J. Y.; Krainer, G.; Lamb, D. C.; Lee, N. K.; Lemke, E. A.; Levesque, B.; Levitus, M.; McCann, J. J.; Naredi-Rainer, N.; Nettels, D.; Ngo, T.; Qiu, R.; Robb, N. C.; Rocker, C.; Sanabria, H.; Schlierf, M.; Schroder, T.; Schuler, B.; Seidel, H.; Streit, L.; Thurn, J.; Tinnefeld, P.; Tyagi, S.; Vandenberk, N.; Vera, A. M.; Weninger, K. R.; Wunsch, B.; Yanez-Orozco, I. S.; Michaelis, J.; Seidel, C. A. M.; Craggs, T. D.; Hugel, T., Precision and accuracy of single-molecule FRET measurements-a multi-laboratory benchmark study. *Nat. Methods* **2018,** *15* (9), 669-676.
4. Kabsch, W.; Sander, C., Dictionary of protein secondary structure: Pattern recognition of hydrogen-bonded and geometrical features. *Biopolymers* **1983,** *22* (12), 2577-2637.
5. Steinhauser, M. O., A molecular dynamics study on universal properties of polymer chains in different solvent qualities. Part I. A review of linear chain properties. *J. Chem. Phys.* **2005,** *122* (9), 094901.
6. Humphrey, W.; Dalke, A.; Schulten, K., VMD: Visual molecular dynamics. *J. Mol. Graph.* **1996,** *14* (1), 33-38.
7. Tesei, G.; Schulze, T. K.; Crehuet, R.; Lindorff-Larsen, K., Accurate model of liquid-liquid phase behavior of intrinsically disordered proteins from optimization of single-chain properties. *Proc. Natl. Acad. Sci. USA* **2021,** *118* (44).
8. Riback, J. A.; Bowman, M. A.; Zmyslowski, A. M.; Plaxco, K. W.; Clark, P. L.; Sosnick, T. R., Commonly used FRET fluorophores promote collapse of an otherwise disordered protein. *Proc. Natl. Acad. Sci. USA* **2019,** *116* (18), 8889-8894.
9. Holmstrom, E. D.; Holla, A.; Zheng, W.; Nettels, D.; Best, R. B.; Schuler, B., Accurate Transfer Efficiencies, Distance Distributions, and Ensembles of Unfolded and Intrinsically Disordered Proteins From Single-Molecule FRET. *Methods Enzymol.* **2018,** *611*, 287-325.
10. Fuertes, G.; Banterle, N.; Ruff, K. M.; Chowdhury, A.; Mercadante, D.; Koehler, C.; Kachala, M.; Estrada Girona, G.; Milles, S.; Mishra, A.; Onck, P. R.; Grater, F.; Esteban-Martin, S.; Pappu, R. V.; Svergun, D. I.; Lemke, E. A., Decoupling of size and shape fluctuations in heteropolymeric sequences reconciles discrepancies in SAXS vs. FRET measurements. *Proceedings of the National Academy of Sciences of the United States of America* **2017,** *114* (31), E6342-E6351.
11. UniProt, C., UniProt: the Universal Protein Knowledgebase in 2023. *Nucleic Acids Res.* **2022**.
12. Tesei, G.; Schulze, T. K.; Crehuet, R.; Lindorff-Larsen, K., Accurate model of liquid-liquid phase behavior of intrinsically disordered proteins from optimization of single-chain properties. *Proceedings of the National Academy of Sciences* **2021,** *118* (44).
13. Tesei, G.; Lindorff-Larsen, K., Improved predictions of phase behaviour of intrinsically disordered proteins by tuning the interaction range. *Open Res Eur* **2022,** *2*, 94.
14. Dannenhoffer-Lafage, T.; Best, R. B., A Data-Driven Hydrophobicity Scale for Predicting Liquid-Liquid Phase Separation of Proteins. *J. Phys. Chem. B* **2021,** *125* (16), 4046-4056.
